# Global burden of Type 2 Diabetes Mellitus attributable to dietary risks in elderly adults: insights from the Global Burden of Disease study 2021

**DOI:** 10.3389/fnut.2025.1557923

**Published:** 2025-04-24

**Authors:** Yiting Tang, Yupeng Chen, Yang Zhou, Bingrong Wu, Shan Zhang, Yanbing Gong, Qing Ni

**Affiliations:** ^1^Beijing University of Chinese Medicine, Beijing, China; ^2^Department of Endocrinology, Guang'anmen Hospital of China Academy of Chinese Medical Sciences, Beijing, China

**Keywords:** Type 2 Diabetes Mellitus, dietary risks, elderly adults, Global Burden 2021, public health interventions, precision nutrition, Socio-Demographic Index

## Abstract

**Background:**

Type 2 Diabetes Mellitus (T2DM) poses a significant global health challenge, particularly among elderly adults. Dietary risk factors, such as high consumption of processed meats and sugar-sweetened beverages and low intake of whole grains and fruits, play a critical role in the burden of T2DM. This study aims to comprehensively quantify the global burden of T2DM attributable to dietary risks among elderly adults, identify significant dietary risk factors driving disease burden, and evaluate temporal, regional, and demographic variations to inform targeted public health strategies and interventions for reducing the impact of T2DM.

**Methods:**

This study utilized data from the Global Burden of Diseases, Injuries, and Risk Factors Study (GBD) 2021 to assess T2DM burden attributable to dietary risks among adults aged 65 years and older across 204 countries and territories. Metrics included age-standardized mortality rates (ASMR) and disability-adjusted life-year rates (ASDR). Dietary risk factors analyzed included low intake of whole grains, fruits, vegetables, and fiber, as well as high intake of processed meat, red meat, and sugar-sweetened beverages. Joinpoint regression and decomposition analyses were performed to examine temporal trends and drivers of changes by region, SDI level, sex, and age.

**Results:**

In 2021, dietary risks accounted for 23.61% of T2DM-related deaths and 24.85% of DALYs among elderly adults. ASMR showed a slight decline globally (AAPC: −0.08), while ASDR exhibited a significant upward trend (AAPC: +0.7) from 1990 to 2021. High SDI regions demonstrated decreasing ASMR but persistent DALYs due to prolonged survival with complications. Conversely, low and middle SDI regions exhibited rapid increases in ASMR and ASDR, driven by dietary transitions and limited healthcare resources. Males consistently bore a higher burden than females, with pronounced disparities in low and middle SDI regions. Aging and population growth were the primary drivers of the increasing burden globally.

**Conclusion:**

This study underscores the substantial burden of T2DM attributable to dietary risks among elderly adults and highlights significant regional and demographic disparities. Targeted public health interventions, personalized nutritional strategies, and improved healthcare access are essential to mitigate this burden. Future research should explore the impact of emerging dietary trends and precision nutrition on T2DM prevention and management.

## 1 Introduction

Diabetes mellitus (DM) is a significant and escalating global public health concern, ranking among the fastest-growing chronic diseases worldwide. By 2021, an estimated 537 million individuals were living with DM, and this figure is projected to rise to 783 million by 2045 (1). Type 2 Diabetes Mellitus (T2DM), which accounts for 90–95% of all cases (2), is most prevalent among individuals aged 65–79 years (3–5), a trend closely associated with global population aging. Among older adults, poor glycemic control contributes to high rates of diabetes-related complications and mortality (6).

Research shows that 50% of diabetic individuals over 65 years develop diabetic nephropathy, characterized by proteinuria or reduced glomerular filtration rates (GFR) (7). Furthermore, older adults with diabetes are at a tenfold higher risk of lower-limb amputation compared to their non-diabetic counterparts (4). As the burden of diabetes in elderly populations continues to rise, the prevention and management of chronic complications remain critical challenges.

The Global Burden of Diseases (GBD), Injuries, and Risk Factors Study identifies several key contributors to diabetes, including environmental and occupational risks, tobacco use, excessive alcohol consumption, high body mass index (BMI), insufficient physical activity, and dietary risks (8). Among these, dietary patterns are particularly influential in the development and progression of T2DM (9, 10). Aging populations face distinct challenges such as metabolic decline, excessive energy intake, increased visceral fat, and sarcopenic obesity (11). Poor dietary habits, including high intake of refined carbohydrates, low dietary diversity, and irregular eating patterns, worsen glycemic control. Conversely, overly restrictive diets may also elevate health risks in elderly diabetic patients (12). Evidence indicates that up to 90% of T2DM cases could be prevented through healthier diets and lifestyles (13). In China, dietary improvements and weight management could prevent 52.7% of T2DM cases in men and 58.4% in women (14).

Studies have shown that high consumption of red and processed meats, low intake of whole grains, inadequate fruit and vegetable consumption, low dietary fiber, and high consumption of sugar-sweetened beverages substantially contribute to the T2DM burden (8). However, the specific impact of dietary risks on elderly populations remains underexplored. Current research often targets general populations or specific regions (15, 16), leaving a critical gap in understanding the global dietary burden of T2DM among older adults.

The growing burden of T2DM among the elderly underscores the need for urgent and targeted public health interventions. Lifestyle changes and medical nutrition therapy are essential strategies for managing and preventing T2DM in this demographic. However, despite recognition of dietary influences on T2DM risk, the specific global burden attributable to dietary risk factors among elderly populations has not been sufficiently quantified. Therefore, the objective of this study is to comprehensively quantify the global burden of T2DM attributable to dietary risks in elderly adults, assess temporal and regional variations from 1990 to 2021, and identify key demographic and socioeconomic drivers of these trends. Through this analysis, the study aims to provide evidence to inform targeted nutritional strategies and public health policies that effectively mitigate the global impact of T2DM in elderly populations.

## 2 Methods

### 2.1 Data source

This study utilized data from the Global Burden of Diseases, Injuries, and Risk Factors Study (GBD) 2021, which evaluates health loss across 371 diseases, injuries, and impairments, and 87 risk factors in 204 countries and territories. The GBD 2021 framework integrates epidemiological data standardized and harmonized through the Global Health Data Exchange (GHDx). To address gaps in data, spatiotemporal Gaussian process regression was used to smooth information across age, time, and geography. Additionally, the Meta-Regression with Bayesian priors, Regularization, and Trimming (MR-BRT) method was applied to adjust for biases related to differences in case definitions and study methodologies. Detailed methods for GBD 2021 are described elsewhere (17). The study adhered to the Guidelines for Accurate and Transparent Health Estimates Reporting (GATHER) (18).

### 2.2 Estimation of T2DM burden

The burden of T2DM in adults aged 65 years and older was assessed using Age-Standardized Mortality Rates (ASMR) and Age-Standardized Disability-Adjusted Life-Year Rates (ASDR), expressed per 100,000 population. The GBD 2021 framework estimates health loss using Disability-Adjusted Life Years (DALYs), which combine Years of Life Lost (YLLs) and Years Lived with Disability (YLDs). T2DM cases were classified using the International Classification of Diseases, Tenth Edition (ICD-10). Details about data sources and metadata are available online at http://ghdx.healthdata.org/gbd-2021/data-input-sources.

### 2.3 Estimation of risk-attributable burden

The burden of T2DM attributable to dietary risk factors was estimated using Population Attributable Fractions (PAFs), which compare actual exposure levels to a theoretical minimum risk level while holding other factors constant. PAFs were applied to calculate the T2DM burden for demographic groups stratified by age, sex, location, and year.

### 2.4 Selection of dietary risk factors

Seven dietary risk factors were analyzed based on their relevance to T2DM progression. Data were retrieved from the GHDx query tool.^1^ The factors included low intake of whole grains, fruits, vegetables, and fiber, as well as high intake of red meat, processed meat, and sugar-sweetened beverages. Each factor was identified as a significant contributor to T2DM risk and progression.

### 2.5 Socio-Demographic Index (SDI)

The Socio-Demographic Index (SDI) was used to assess socioeconomic influences on T2DM burden. SDI is a composite measure incorporating fertility rates in individuals under 25 years, average educational attainment for those aged 15 years and older, and per capita income adjusted for inflation (19). Regions were categorized into five SDI levels: high, high-middle, middle, low-middle, and low.

### 2.6 Decomposition analysis

Decomposition analysis was performed to identify factors contributing to changes in T2DM burden attributable to dietary risks from 1990 to 2021. Changes in mortality and DALYs were separated into components reflecting population growth, age structure changes, exposure to risk factors, and residual factors such as risk-deleted rates.

### 2.7 Statistical analysis

T2DM burden attributable to dietary risks was assessed using deaths, DALYs, and Age-Standardized Rates (ASRs), with 95% Uncertainty Intervals (UIs). Joinpoint regression analysis was applied to segment temporal trends, identify inflection points, and calculate Annual Percentage Change (APC) with 95% Confidence Intervals (CIs) (20). Average Annual Percentage Change (AAPC) was derived by weighting segment-specific APCs by duration. A Monte Carlo permutation method (4,499 iterations) ensured statistical significance, corrected with Bonferroni adjustment (21). Trends were classified as increasing if the lower bound of the 95% CI exceeded zero, decreasing if the upper bound was below zero, or stable if neither condition was met.

## 3 Results

### 3.1 Global trends in T2DM burden attributable to dietary risk factors in elderly adults (1990–2021)

Analysis of GBD 2021 data showed that, in 2021, dietary risk factors accounted for 23.61% (95% UI: 18.73–30.45) of T2DM-related mortality and 24.85% (95% UI: 18.18–30.53) of T2DM-related DALYs in elderly adults. These proportions declined from 26.53% (95% UI: 21.98–32.98) and 26.58% (95% UI: 22.11–33.51), respectively, in 1990. The contributions of specific dietary factors varied over time. Diets high in processed meat were notable contributors in 1990, accounting for 9.0% of deaths and DALYs, but these decreased to 7.2 and 8.3% by 2021. Similarly, diets high in red meat showed minor changes, remaining steady at 4.3–4.9%. Conversely, sugar-sweetened beverage consumption exhibited a rising burden, increasing from 2.8% in 1990 to 3.1% (deaths) and 3.5% (DALYs) in 2021 (Supplementary material 1).

In 2021, the ASMR and ASDR for diet-related T2DM were 36.16 (95% UI: 6.95–59.35) and 1,091.50 (95% UI: 229.37–1,835.49) per 100,000 population, respectively. While ASMR exhibited a slight decline (AAPC: −0.08; 95% CI: −0.22 to 0.06), ASDR demonstrated a significant upward trend (AAPC: +0.7; 95% CI: +0.63 to +0.77) between 1990 and 2021 (Supplementary material 2). Major contributors to ASMR and ASDR included diets high in processed and red meat and low in whole grains (Supplementary material 2).

**Figure 1:**
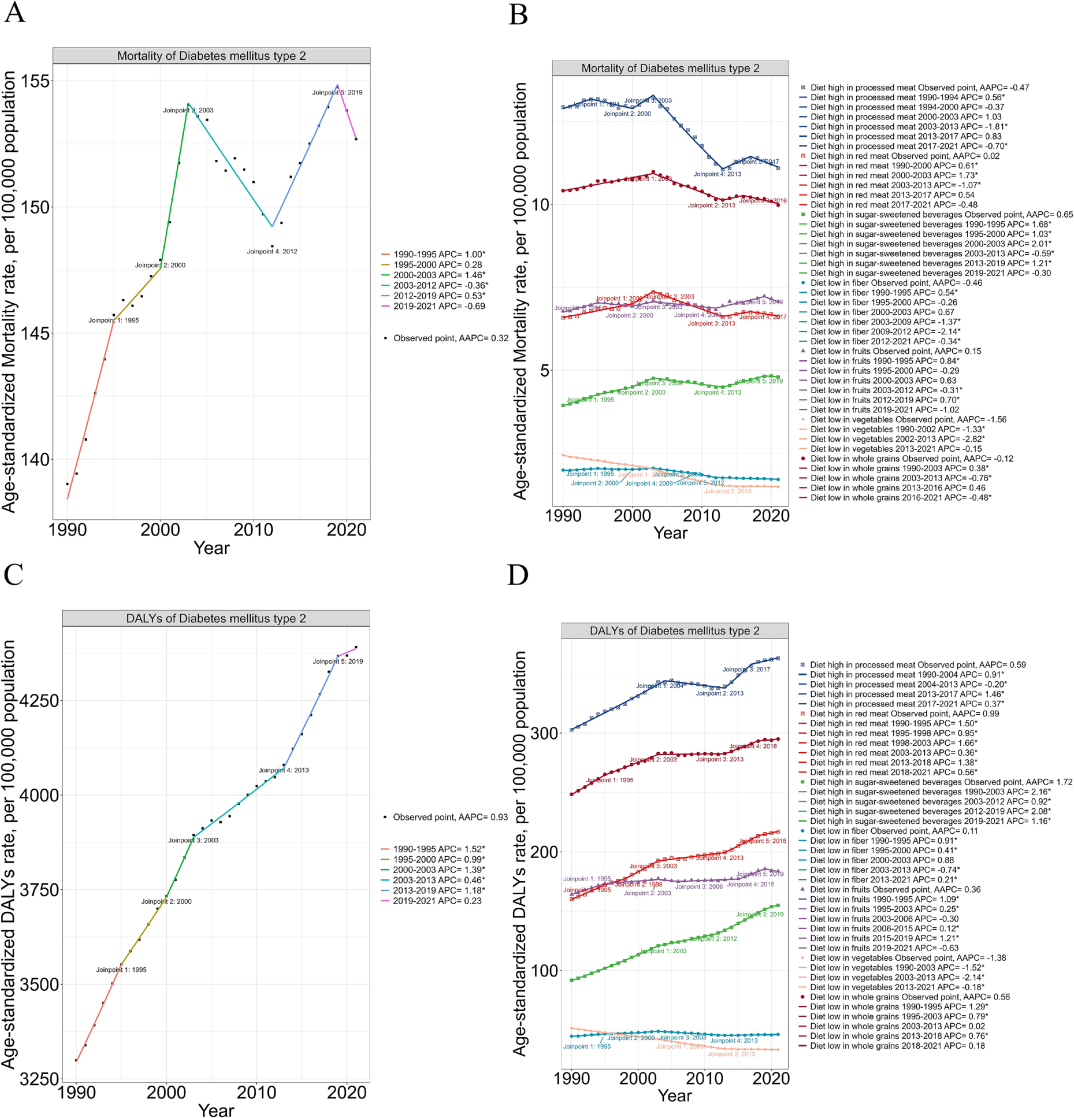
Trends in Type 2 Diabetes Mellitus mortality and DALYs attributable to dietary risks in elderly adults, 1990–2019. **(A)** Age-standardized mortality rates (ASMR) of Type 2 Diabetes Mellitus (T2DM) from 1990 to 2019, showing joinpoint analysis with significant trends. Each segment indicates periods of change in the Average Annual Percentage Change (AAPC). **(B)** Breakdown of T2DM mortality by specific dietary risks, including high consumption of processed meat, red meat, sugar-sweetened beverages, and low consumption of whole grains and vegetables. Joinpoint analysis highlights critical transition years for dietary risk trends. **(C)** Age-standardized disability-adjusted life years (ASDR) for T2DM from 1990 to 2019. The trend shows a consistent increase, with marked points of statistical joinpoints indicating shifts in AAPC. **(D)** Detailed analysis of dietary risk factors associated with T2DM DALYs. The graph delineates the impact of specific dietary risks on ASDR, with notable shifts in trends identified through joinpoint regression. ASMR and ASDR are expressed per 100,000 population. Dietary risks include consumption of processed meat, red meat, sugar-sweetened beverages, and insufficient intake of whole grains, fruits, and vegetables. Joinpoint regression analysis identifies significant periods of increasing or decreasing trends, represented by distinct color-coded segments. Data are derived from the Global Burden of Disease (GBD) Study 2021.

Globally, ASMR trends fluctuated. Between 1990 and 1995, ASMR increased significantly (APC: +1.00), slowed between 1995 and 2000 (APC: +0.28), and rose sharply from 2000 to 2003 (APC: +1.46). A decline followed from 2003 to 2012 (APC: −0.36), rebounded modestly from 2012 to 2019 (APC: +0.53), and declined again after 2019 (APC: −0.69) (Figure 1A). ASDR, in contrast, consistently rose with an overall AAPC of +0.58. After steady increases between 1990 and 2003, ASDR grew at a slower pace from 2003 to 2013 (APC: +0.46), accelerated from 2012 to 2019 (APC: +1.18), and remained positive but slowed after 2019 (APC: +0.23) (Figure 1C). The burden of high processed and red meat intake declined slightly after the early 2000s, while sugar-sweetened beverage consumption consistently increased, reflecting global dietary shifts. Meanwhile, risks associated with low vegetable and fiber intake gradually decreased (Figures 1B,D).

### 3.2 Regional trends in the dietary risk-attributable burden of T2DM in elderly adults

**Figure 2:**
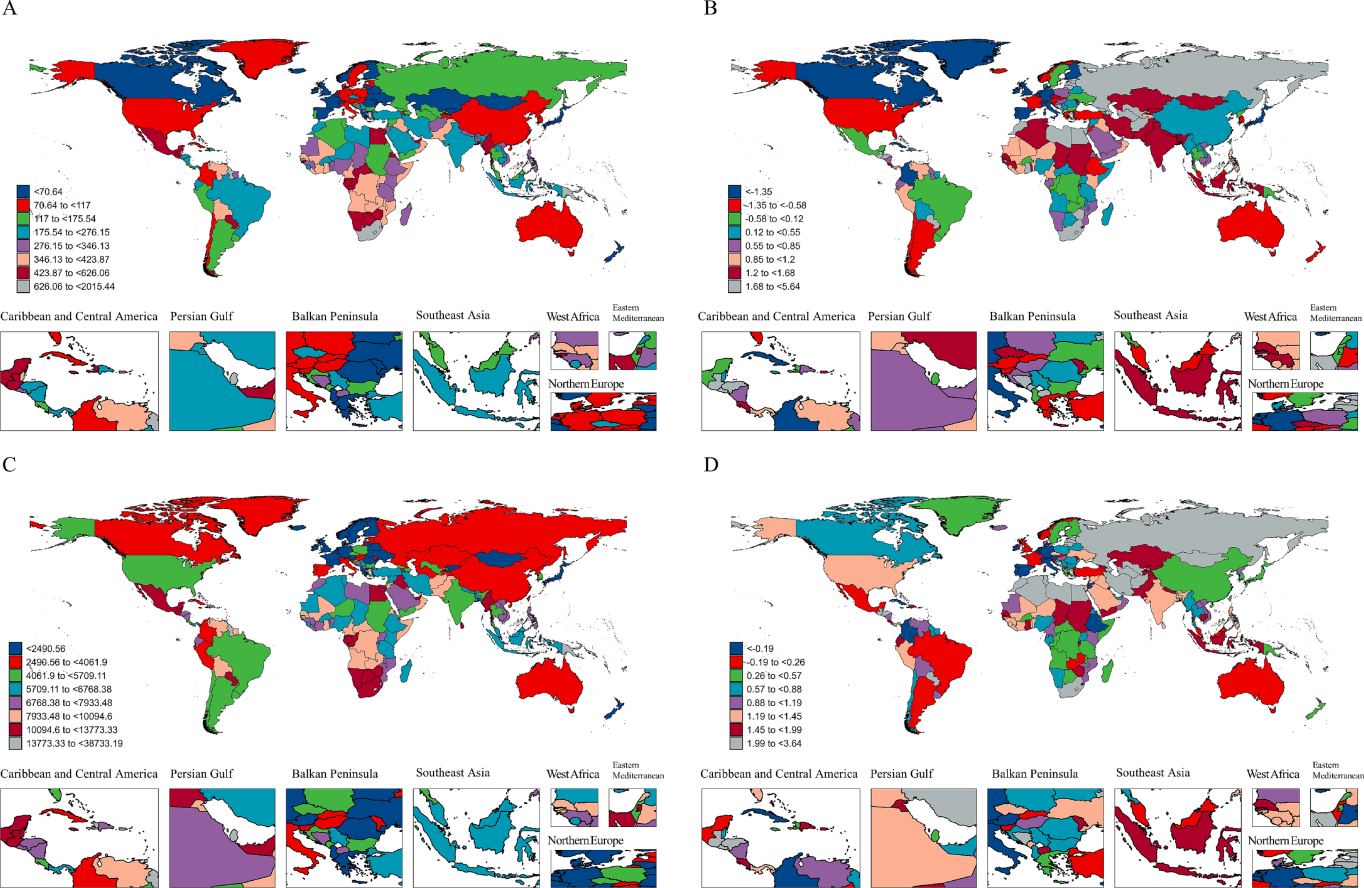
Global distribution and trends of mortality and DALYs attributable to Type 2 Diabetes Mellitus (T2DM) dietary risks in elderly adults. **(A)** Global map showing the age-standardized mortality rate (ASMR) for T2DM attributable to dietary risks in 2021. The color gradient represents the severity, with red indicating the highest ASMR and blue the lowest. **(B)** Average annual percentage change (AAPC) in ASMR from 1990 to 2021. Positive values indicate an increasing trend, while negative values represent a decreasing trend over the period. Regional differences highlight variable progress in controlling dietary risks related to T2DM mortality. **(C)** Global map of age-standardized DALYs rate (ASDR) for T2DM in 2021. Similar to the ASMR map, the color gradient illustrates the burden, with red showing the highest ASDR and blue showing the lowest. **(D)** AAPC in ASDR from 1990 to 2021, illustrating temporal trends in the burden of T2DM attributable to dietary risks. Increasing trends (positive AAPC) are shown in red, while decreasing trends (negative AAPC) are shown in blue. Regions include subpanels for focused areas like the Caribbean, Persian Gulf, Balkan Peninsula, Southeast Asia, West Africa, and Northern Europe to provide detailed regional insights.

The burden of T2DM attributable to dietary risks showed substantial regional variations between 1990 and 2021 (Supplementary material 2; Figure 2). High-income regions, such as Western Europe and North America, experienced declining ASMR trends driven by reductions in processed and red meat consumption. For instance, Western Europe’s ASMR related to processed meat declined (AAPC: −1.7%). In contrast, low-and middle-income regions, such as Sub-Saharan Africa, South Asia, and Southeast Asia, demonstrated significant increases in ASMR, particularly from sugar-sweetened beverages and insufficient fiber intake. Southeast Asia had the highest global AAPC for sugar-sweetened beverage-related mortality (+3.74%).

ASDR trends reflected similar disparities. While high-income regions like Australasia and North America exhibited stabilization or modest increases, Central Asia, the Middle East, and Sub-Saharan Africa showed rapid ASDR growth. For example, ASDR attributable to processed meat in Central Asia rose (AAPC: +2.49%), mirroring dietary transitions. Low whole grain intake also significantly contributed to ASDR in Central Asia (AAPC: +2.41%) and the Middle East (AAPC: +1.36%).

In low-income regions, low fruit and vegetable consumption remained key contributors. Sub-Saharan Africa’s ASDR attributable to low fruit intake increased (AAPC: +1.4%), while South Asia showed rising burdens from sugar-sweetened beverages (mortality AAPC: +3.56%, ASDR AAPC: +3.4%). Conversely, Central and Eastern Europe, while improving fruit and vegetable intake, faced continued burdens from processed and red meat consumption, as indicated by rising ASDR.

### 3.3 Global trends in T2DM burden attributable to dietary risk factors by age

**Figure 3:**
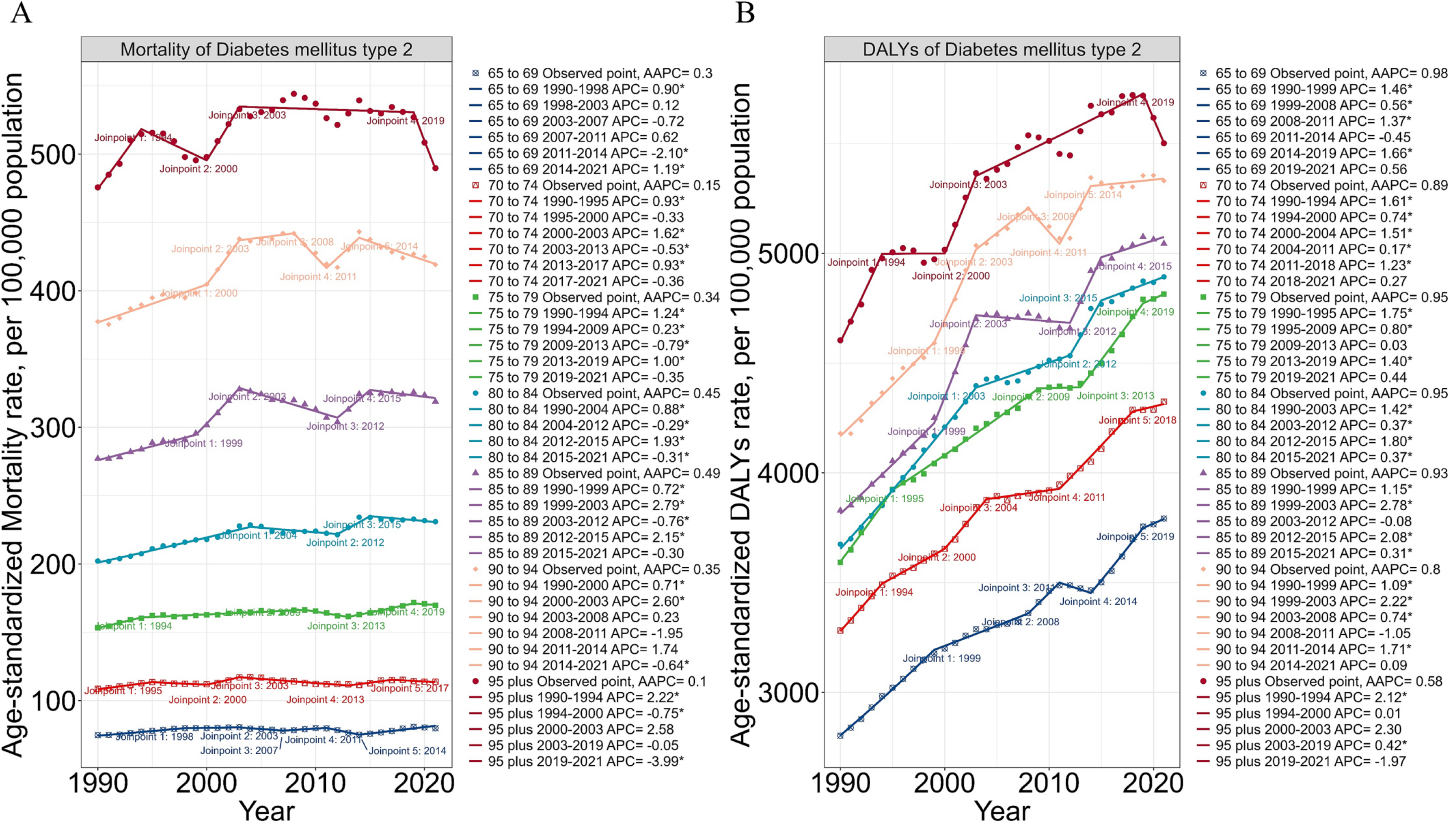
Age-specific trends in mortality and DALYs of Type 2 Diabetes Mellitus (T2DM) attributable to dietary risks in elderly adults. **(A)** Trends in crude mortality rates for T2DM, stratified by age groups (65–69, 70–74, 75–79, 80–84, 85–89, and 90 + years) from 1990 to 2021. Joinpoint regression analysis highlights significant shifts in Average Annual Percentage Change (AAPC) for each age group, represented by distinct markers and segmented lines. **(B)** Crude disability-adjusted life years (DALYs) rate trends for T2DM, stratified by the same age groups as in panel **(A)**. Joinpoint analysis captures variations in trends over time, with segments indicating periods of significant change in AAPC. Each line represents a specific age group, with data points indicating observed rates and joinpoints marking statistically significant changes in trends. AAPC values are provided for each age group, reflecting long-term shifts in mortality and DALY rates. Data reveal an increasing burden of T2DM among older age groups, particularly in DALYs, underlining the growing impact of dietary risks in aging populations.

Joinpoint regression analysis revealed significant age-specific trends in T2DM burden attributable to dietary risks among elderly adults aged 65 and above (Figure 3). Crude mortality rates (CMR) showed both increasing and fluctuating patterns, with notable shifts at key time points. For younger elderly groups, such as 65–69 and 70–74 years, early increases in CMR were followed by stabilization and eventual declines, particularly after 2011 for the 65–69 group (APC: −2.10). Older age groups, including 85–89 and 90–94 years, experienced more pronounced increases, with rapid rises during 1999–2003 (APC: +2.79) and 2000–2003 (APC: +2.60), respectively. The 95 + age group showed a unique pattern of early growth in mortality (1990–1994 APC: +2.22) followed by a marked decline after 2019 (APC: −3.99).

Crude DALY rates (CDR) demonstrated a consistent upward trajectory across all elderly age groups. The steepest increases were observed in individuals aged 85 and above, with significant shifts in growth noted at joinpoints in 1994, 2003, and 2015, highlighting the cumulative impact of dietary risks over a lifetime. Similarly, the 70–74 and 75–79 age groups experienced rapid rises in CDR, with joinpoints in 2011 and 2013, coinciding with global dietary transitions, such as increased consumption of processed foods and sugar-sweetened beverages. These findings indicate that while younger elderly groups encountered earlier transitions in T2DM burden, older populations have exhibited sustained growth in both mortality and DALYs over time.

### 3.4 Global trends in T2DM burden attributable to dietary risk factors by sex

**Figure 4:**
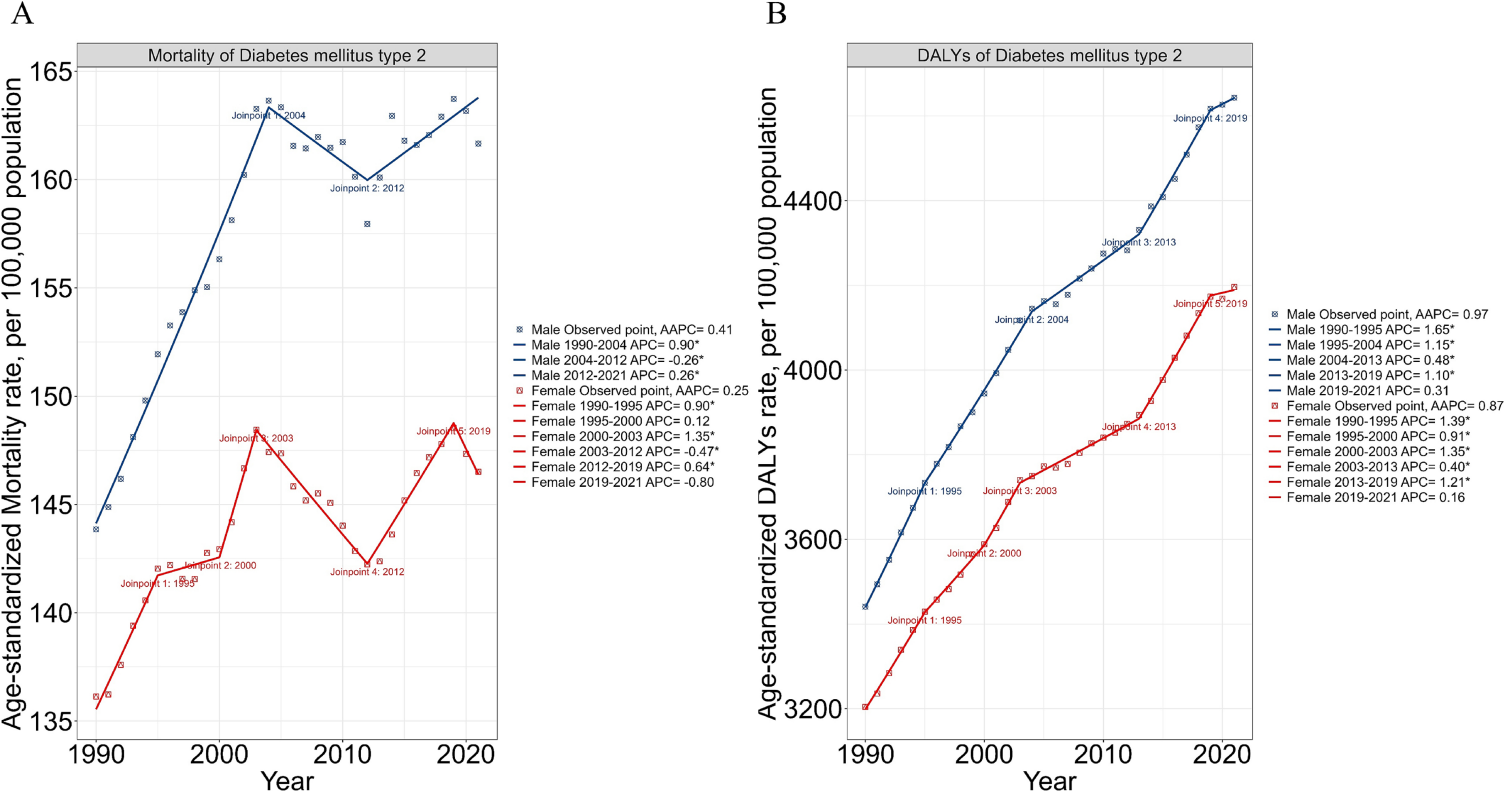
Gender-specific trends in mortality and DALYs of Type 2 Diabetes Mellitus (T2DM) attributable to dietary risks in elderly adults. **(A)** Age-standardized mortality rates (ASMR) for males and females from 1990 to 2021. The blue line represents males, while the red line represents females. Joinpoint regression analysis reveals significant shifts in trends, with periods of increasing or decreasing Average Annual Percentage Change (AAPC) indicated by distinct line segments. **(B)** Age-standardized disability-adjusted life years (ASDR) for males and females over the same period. Trends are illustrated with blue (males) and red (females) lines, showcasing consistent increases for males and more variable trends for females. Joinpoints highlight statistically significant changes in AAPC. Blue and red data points correspond to observed values for males and females, respectively. Males generally exhibit higher ASMR and ASDR rates compared to females, highlighting gender differences in the burden of T2DM attributable to dietary risks.

Distinct sex-specific patterns emerged in the burden of T2DM attributable to dietary risks between 1990 and 2021 (Figure 4). For males, ASMR increased steadily from 1990 to 2004 (APC: +0.90), then declined slightly between 2004 and 2012 (APC: −0.26). From 2012 to 2021, ASMR resumed growth at a slower rate (APC: +0.26). Similarly, ASDR attributable to dietary risks for males rose consistently throughout the study period, peaking from 1990 to 1995 (APC: +1.65), with growth rates slowing between 2004 and 2013 (APC: +0.48) and stabilizing from 2019 to 2021 (APC: +0.31). Females exhibited more variable trends in both ASMR and ASDR. ASMR for females rose rapidly between 1990 and 1995 (APC: +0.90), then declined between 2003 and 2012 (APC: −0.47). Mortality rates increased significantly from 2012 to 2019 (APC: +0.64), before declining slightly post-2019 (APC: −0.80). ASDR for females showed notable increases during 1990–1995 (APC: +1.39), 2000–2003 (APC: +1.35), and 2013–2019 (APC: +1.21), with stabilization observed from 2019 to 2021 (APC: +0.16).

Sex-specific differences were also evident in the contributions of individual dietary risks (Table 1). High consumption of processed and red meats showed a modestly increasing trend in ASDR for males (AAPC: +0.81), while females exhibited stabilization or smaller increases (AAPC: +0.60). Males consistently showed higher ASDR rates for diets high in red meat (AAPC: +1.29) and sugar-sweetened beverages (AAPC: +2.05) compared to females. Conversely, risks such as low fiber and vegetable intake showed consistent declines in ASMR for both sexes, with males experiencing sharper reductions (AAPC for low vegetables: −2.10 in males vs. −1.10 in females). Diets low in whole grains revealed slight increases in ASDR for both sexes, with males demonstrating higher rates and faster annual growth (AAPC: +0.68 for males vs. +0.40 for females). Overall, males bore a consistently higher burden in both ASMR and ASDR throughout the study period.

**Table 1:** Global age-standardized rates and average annual percent changes attributable to 7 dietary factors for T2DM burden attributable to dietary risk factors in elderly adults by sex, 1990 and 2019.

Dietary factors	Sex	Age-standardized rate per 100,000 people (95% UI)				Average annual percent change from 1990 to 2021(95% CI)	
		1990		2021			
		Death rate	DALYs rate	Death rate	DALYs rate	Death rate	DALYs rate
Diet high in processed meat	Both	12.92 (3.06–21.17)	302.67 (71.95–510.06)	11.09 (2.59–18.43)	362.95 (85.28–625.45)	−0.47 (−0.69 to −0.25)	0.59 (0.51 to 0.66)
	Female	13.45 (3.18–22.04)	310.15 (73.61–521.89)	10.92 (2.56–18.28)	353.51 (83.14–611.26)	−0.66 (−0.87 to −0.44)	0.43 (0.33 to 0.52)
	Male	12.04 (2.86–19.72)	291.11 (69.50–491.91)	11.30 (2.62–18.74)	374.91 (87.99–646.53)	−0.21 (−0.38 to −0.05)	0.82 (0.77 to 0.88)
Diet high in red meat	Both	6.59 (−0.95–14.74)	160.24 (−24.17–360.88)	6.62 (−0.92–14.86)	216.99 (−33.11–498.27)	0.02 (−0.17 to 0.22)	0.99 (0.88 to 1.1)
	Female	6.84 (−0.98–15.29)	163.57 (−24.62–367.91)	6.29 (−0.89–14.17)	205.84 (−31.16–473.23)	−0.25 (−0.46 to −0.04)	0.75 (0.63 to 0.87)
	Male	6.18 (−0.89–13.79)	155.33 (−23.51–350.59)	7.06 (−0.98–16.01)	231.01 (−35.80–528.08)	0.46 (0.25 to 0.67)	1.29 (1.18 to 1.4)
Diet high in sugar-sweetened beverages	Both	3.94 (1.97–5.94)	91.87 (45.71–142.35)	4.80 (2.41–7.11)	154.98 (78.22–238.14)	0.65 (0.46 to 0.83)	1.72 (1.67 to 1.76)
	Female	4.26 (2.07–6.55)	97.61 (46.67–152.68)	4.77 (2.39–7.21)	154.05 (76.69–241.41)	0.38 (0.16 to 0.6)	1.5 (1.44 to 1.56)
	Male	3.44 (1.73–5.32)	83.50 (41.85–130.06)	4.82 (2.38–7.23)	156.16 (77.30–243.83)	1.11 (0.96 to 1.26)	2.05 (2 to 2.1)
Diet low in fiber	Both	1.99 (1.12–2.85)	44.72 (25.03–64.87)	1.71 (0.96–2.49)	46.23 (25.45–68.54)	−0.46 (−0.63 to −0.29)	0.11 (0.01 to 0.22)
	Female	1.97 (1.12–2.83)	43.78 (24.70–63.75)	1.70 (0.94–2.50)	45.64 (24.78–68.68)	−0.44 (−0.64 to −0.24)	0.14 (0.04 to 0.24)
	Male	2.04 (1.13–2.94)	46.20 (25.28–67.14)	1.73 (0.96–2.55)	47.07 (25.51–71.48)	−0.53 (−0.62 to −0.44)	0.06 (−0.01 to 0.13)
Diet low in fruits	Both	6.78 (1.09–12.11)	164.08 (25.63–296.03)	7.07 (1.09–12.46)	183.64 (28.33–332.18)	0.15 (−0.06 to 0.36)	0.36 (0.25 to 0.47)
	Female	6.38 (1.02–11.39)	155.31 (24.08–280.77)	6.79 (1.07–12.06)	175.91 (27.89–321.80)	0.21 (0.05 to 0.37)	0.4 (0.33 to 0.48)
	Male	7.44 (1.19–13.35)	177.27 (27.72–325.47)	7.49 (1.10–13.12)	193.71 (28.87–344.79)	0.06 (−0.07 to 0.18)	0.3 (0.23 to 0.38)
Diet low in vegetables	Both	2.44 (−0.91–5.22)	51.69 (−19.40–110.64)	1.49 (−0.56–3.30)	33.57 (−12.70–74.45)	−1.56 (−1.64 to −1.48)	−1.38 (−1.43 to −1.33)
	Female	2.03 (−0.77–4.40)	43.10 (−16.53–93.87)	1.42 (−0.54–3.21)	31.88 (−12.18–71.84)	−1.1 (−1.24 to −0.96)	−0.97 (−1.06 to −0.88)
	Male	3.10 (−1.13–6.78)	64.39 (−23.89–138.71)	1.60 (−0.57–3.54)	36.00 (−13.39–80.61)	−2.1 (−2.33 to −1.87)	−1.85 (−1.96 to −1.74)
Diet low in whole grains	Both	10.41 (3.02–17.01)	248.45 (72.14–419.18)	9.98 (2.79–16.36)	295.04 (83.09–502.57)	−0.12 (−0.25 to 0.01)	0.56 (0.51 to 0.61)
	Female	9.78 (2.78–16.10)	231.55 (66.16–389.91)	8.90 (2.50–14.70)	262.19 (73.49–449.80)	−0.29 (−0.42 to −0.16)	0.4 (0.34 to 0.46)
	Male	11.40 (3.39–18.83)	272.67 (80.15–460.84)	11.46 (3.20–18.87)	336.43 (95.47–574.10)	0.05 (−0.02 to 0.13)	0.68 (0.6 to 0.76)
Dietary risk	Both	37.27 (7.09–60.39)	881.16 (170.00–1461.83)	36.16 (6.95–59.35)	1091.50 (229.37–1835.49)	−0.08 (−0.22 to 0.06)	0.7 (0.63 to 0.77)
	Female	36.87 (6.97–59.75)	863.37 (166.15–1424.52)	34.46 (6.77–56.76)	1036.31 (221.09–1745.56)	−0.2 (−0.36 to −0.03)	0.6 (0.51 to 0.68)
	Male	37.87 (7.37–61.86)	906.66 (175.91–1517.47)	38.52 (7.27–63.19)	1161.82 (240.78–1960.05)	0.05 (−0.06 to 0.16)	0.81 (0.73 to 0.88)

### 3.5 Global trends in T2DM burden attributable to dietary risk factors by SDI levels

**Figure 5:**
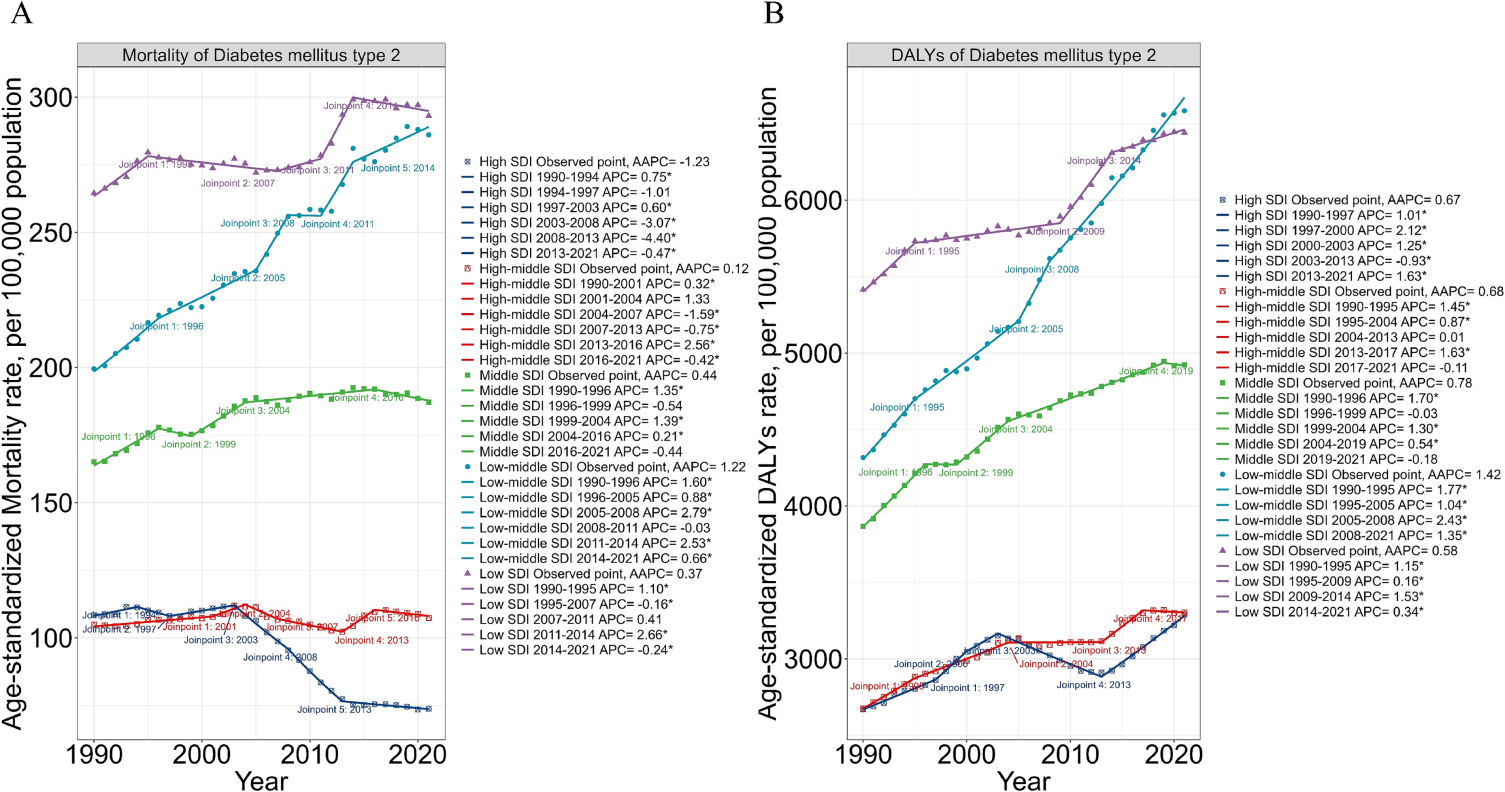
Trends in mortality and DALYs of Type 2 Diabetes Mellitus (T2DM) attributable to dietary risks in elderly adults across Socio-Demographic Index (SDI) Levels. **(A)** Age-standardized mortality rates (ASMR) of T2DM from 1990 to 2021, stratified by SDI levels (High SDI, High-middle SDI, Middle SDI, Low-middle SDI, and Low SDI). Joinpoint regression analysis highlights periods of significant changes in trends, with AAPC values shown for each SDI category. **(B)** Age-standardized disability-adjusted life years (ASDR) rates for T2DM over the same period, stratified by SDI levels. The trajectories of DALYs exhibit significant disparities across SDI categories, with joinpoint analyses marking shifts in AAPC. ASMR and ASDR are expressed per 100,000 population. Color-coded lines represent SDI levels: Blue: High SDI. Purple: High-middle SDI. Green: Middle SDI. Cyan: Low-middle SDI. Red: Low SDI. The burden of T2DM attributable to dietary risks is higher in lower SDI regions, reflecting disparities in dietary risk factor exposure and healthcare access.

The burden of T2DM attributable to dietary risk factors showed significant variation across SDIlevels, with distinct temporal trends marked by joinpoints. In high SDI regions, ASMR for dietary risks consistently declined from 1990 to 2021, with accelerated reductions noted in the early 2000s (Figure 5A). For example, ASDR linked to diets high in processed meat decreased by −1.32% annually (95% CI: −1.56 to −1.09). However, ASDR associated with sugar-sweetened beverages increased annually by 0.75% (95% CI: 0.67 to 0.83). This divergence highlights a scenario where reductions in mortality are accompanied by increased years lived with disability. Sugar-sweetened beverages have emerged as a growing concern in these regions, with ASDR rising sharply at an annual rate of 1.68% (95% CI: 1.50 to 1.86), especially after 2005 (Supplementary material 2).

Conversely, middle, low-middle, and low SDI regions exhibited upward trends in both ASMR and ASDR for nearly all dietary risks, with joinpoints indicating periods of significant change (Figure 5B). For example, in low SDI regions, ASDR attributable to sugar-sweetened beverages increased rapidly after 2000, with annual growth of 1.36% (95% CI: 1.31 to 1.42). Low-middle SDI regions also saw pronounced increases in ASDR for processed meat and sugar-sweetened beverages, particularly post-2010 (Supplementary material 2). Similar trends were observed in middle SDI regions, where ASDR for sugar-sweetened beverages rose sharply by 2.19% annually (95% CI: 1.94 to 2.45) since the early 2000s. While high and middle SDI regions experienced declines in ASMR linked to low intake of fruits, vegetables, and fiber, these reductions were less pronounced in lower SDI regions (Supplementary material 2). These findings emphasize the need for tailored dietary interventions, particularly in low-income regions undergoing rapid dietary transitions, to curb the growing T2DM burden.

### 3.6 Drivers of changes in risk-attributable deaths and DALYs

**Figure 6:**
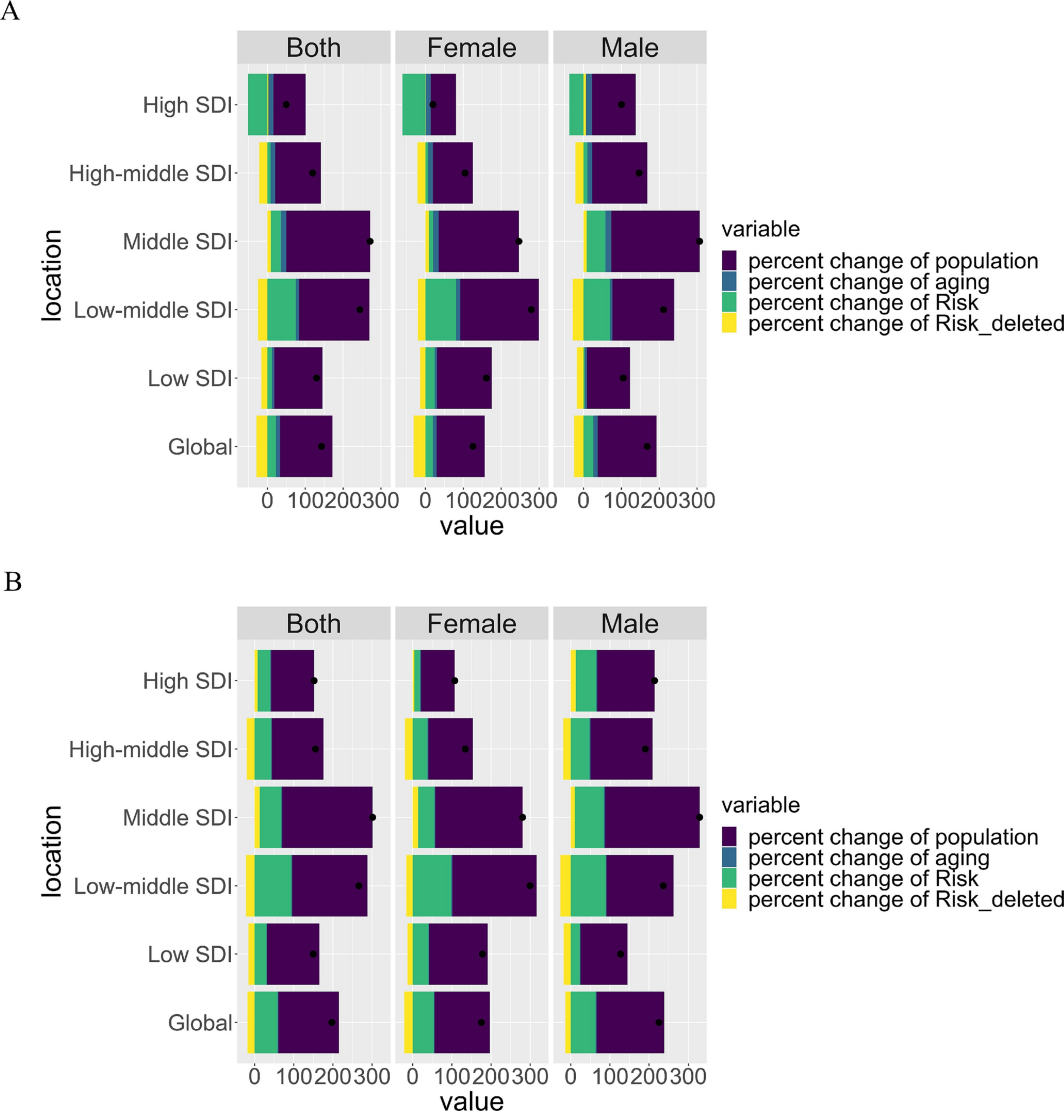
Contributions of population growth, aging, and risk changes to mortality and DALYs of Type 2 Diabetes Mellitus (T2DM) in elderly adults across SDI Levels. **(A)** Decomposition of factors contributing to the percent change in T2DM mortality attributable to dietary risks from 1990 to 2021. Bar plots represent contributions from population growth (purple), aging (green), changes in dietary risk exposure (yellow), and residuals (teal). The data is stratified by gender (Both, Female, Male) and SDI levels (Global, High SDI, High-middle SDI, Middle SDI, Low-middle SDI, and Low SDI). **(B)** Similar decomposition analysis for T2DM disability-adjusted life years (DALYs) attributable to dietary risks during the same period. The contribution of each factor is visualized using the same color scheme, allowing for a comparison of their relative impacts across SDI levels and gender. Purple bars: percent change due to population growth. Green bars: percent change due to population aging. Yellow bars: percent change due to shifts in dietary risk exposure. Teal bars: residual factors not captured by the primary drivers. The charts highlight disparities across SDI levels, with low-SDI regions experiencing greater contributions from population growth and risk exposure compared to high-SDI regions. Black dots indicate the total percent change attributable to all factors combined, providing a cumulative perspective for each group.

Figure 6 presents the relative contributions of population growth, aging, and dietary risk exposure to global and regional trends in T2DM-related deaths and DALYs from 1990 to 2021. Globally, population growth was the primary driver of the rising T2DM burden, particularly in low and low-middle SDI regions. Aging contributed significantly in high and high-middle SDI regions, where its impact surpassed that of dietary risks. Changes in dietary risk exposure had a moderate but notable influence, particularly in low and middle SDI regions, where improvements in dietary management significantly mitigated the T2DM burden.

The influence of dietary risk exposure varied across SDI levels. High and middle SDI regions experienced reductions in exposure to certain risks, such as high processed meat consumption, which partially offset increases in deaths and DALYs. In contrast, low and low-middle SDI regions faced rising exposures to high-risk diets, particularly sugar-sweetened beverages and low fiber intake, contributing substantially to the T2DM burden. These findings underscore regional disparities in the drivers of the T2DM burden and the critical need for region-specific strategies to address dietary risk factors (Supplementary material 3).

## 4 Discussion

Our analysis of the GBD data provides an in-depth evaluation of the global burden of T2DM attributable to dietary risk factors among elderly adults from 1990 to 2021. The findings reveal a notable rise in ASMR and ASDR during this period, largely driven by dietary factors such as insufficient intake of whole grains and fruits, alongside excessive consumption of sugar-sweetened beverages and processed meats. Marked geographical disparities were identified, with Central Asia and Southern Sub-Saharan Africa recording the highest annual increases in ASMR and ASDR. The study also highlights the interplay between SDI levels and the T2DM burden. Low and low-middle SDI regions experienced rapid growth in dietary risk-attributable DALYs, reflecting ongoing dietary transitions and limited healthcare resources. In contrast, high SDI regions demonstrated stabilization or modest growth in burden, suggesting that improved healthcare and dietary practices may be mitigating some risks. These findings underscore the need for targeted interventions to address dietary risks, particularly in vulnerable regions, to curb the rising global impact of T2DM.

Dietary factors play a crucial role in the prevention and management of T2DM. Diets rich in whole grains and leafy vegetables, combined with reduced consumption of refined grains, processed meats, red meat, and sugar-sweetened beverages, have been shown to significantly lower the risk of developing T2DM (22–24). In 2021, dietary risks accounted for 23.61% of T2DM-related deaths among individuals aged 65 years and older worldwide, second only to overweight and obesity. These dietary risks not only directly contribute to T2DM but also increase the prevalence of overweight and obesity, further compounding the likelihood of disease development (25). Our findings corroborate the pivotal role of dietary factors, particularly diets high in processed and red meats and low in whole grains, in exacerbating the T2DM burden. This aligns with global trends in nutritional epidemiology (26). The adverse effects of processed and red meat consumption on T2DM are mediated through several mechanisms, including heightened inflammation and oxidative stress (27), DNA damage induced by additives like nitrites (28), and insulin resistance caused by heme iron and saturated fatty acids (29). Additional contributors, such as excessive protein intake (30) and endotoxin effects (31), further aggravate chronic inflammatory responses and metabolic dysfunction. Aging amplifies these adverse effects by impairing metabolic regulation, thereby intensifying the detrimental impact of unhealthy dietary patterns in elderly individuals. Furthermore, globalization and urbanization have driven shifts toward energy-dense processed foods, disproportionately affecting low-and middle-income regions, which now bear the highest proportional burden of dietary risk-related T2DM (32). These insights highlight the urgent need for dietary interventions focused on modifiable risk factors, particularly in vulnerable populations. Implementing strategies to promote healthier dietary patterns can play a key role in mitigating the escalating global diabetes epidemic.

The gender and age distribution of T2DM deaths attributable to dietary risk factors in our study reveals older adults, particularly those aged 75 and above, experience disproportionately higher ASMR and ASDR compared to younger elderly groups. This can be attributed to age-related metabolic changes, including decreased insulin sensitivity and exacerbated dietary risk exposures, which are consistent with findings by Guan et al. (33). Age-associated increases in chronic inflammation and oxidative stress exacerbate insulin resistance (34), while pancreatic *β*-cell dysfunction and impaired glucose tolerance further heighten diabetes risk (35). Additionally, visceral fat accumulation, which occurs across all ethnicities with aging and is independent of overall weight gain, contributes significantly to the T2DM burden (36). Older adults also face challenges in altering entrenched dietary habits, compounded by age-related declines in health literacy and self-management abilities, which further complicate disease management (37). Notable sex differences were observed, with males consistently experiencing a higher burden of T2DM-related mortality and disability than females. Between 2003 and 2012, both genders exhibited declines in ASMR, attributed to the adoption of standardized diabetes treatment guidelines and nationwide health campaigns promoting healthy eating. For example, the United States’ Diabetes Prevention Program (DPP) emphasized lifestyle modifications and effectively reduced the overall disease burden (38). However, after 2012, ASMR trends diverged, with rates for males increasing and those for females declining starting in 2019. These disparities may be influenced by behavioral differences, such as higher red and processed meat consumption and lower adherence to protective dietary patterns among males (39). Females, on the other hand, may have benefited more from preventive interventions due to greater engagement with healthcare systems (40). Additionally, sex-specific epigenetic mechanisms and hormonal variations, as highlighted by Danquah et al. (41), likely play a role in modulating T2DM risk and progression. These findings underscore the necessity of age-and sex-specific prevention and management strategies to address the growing global T2DM burden in elderly populations. Tailored interventions considering metabolic, behavioral, and physiological differences are crucial to mitigating this public health challenge.

Disparities in the burden of T2DM attributable to dietary risk factors among elderly adults vary significantly across SDI levels and geographic regions. High SDI regions, such as Western Europe and North America, report relatively lower ASMR and ASDR. Since 2003, these regions have shown consistent declines in ASMR, reflecting the impact of healthier dietary habits and cultural norms promoting high-quality diets (42). Diets in these areas are typically rich in fruits, vegetables, and whole grains (43). However, despite lower mortality rates, high SDI regions continue to face significant DALYs due to increased survival among elderly individuals with long-term complications, prolonging exposure to disability. In contrast, middle and low SDI regions demonstrate a growing T2DM burden attributable to dietary risks, driven by rapid urbanization, increased consumption of processed foods, and limited healthcare access. Dietary patterns in low SDI regions are shaped by economic constraints, favoring energy-dense, nutrient-poor foods. For instance, South Asia and Sub-Saharan Africa exhibit the highest ASMR and ASDR among elderly populations. These trends align with findings by Shao et al. (44), who observed similar patterns in chronic conditions exacerbated by dietary transitions. Low SDI countries often struggle with inadequate healthcare infrastructure, low health literacy, and limited preventive care, leading to disproportionately higher mortality and DALYs from dietary risks. Addressing these disparities requires targeted interventions to improve access to healthier diets, enhance public health education, and strengthen healthcare systems in resource-constrained settings.

The decomposition analysis of changes in the burden of T2DM attributable to dietary risk factors by SDI and sex reveals substantial regional and demographic differences. Population growth and aging are the primary drivers of the increasing global T2DM burden, with particularly pronounced effects in low and middle SDI regions where limited healthcare access and demographic shifts exacerbate risk factors. In high and high-middle SDI regions, population aging is the predominant contributor, reflecting the combined impact of longer life expectancy and cumulative dietary risk exposure. These findings align with prior research highlighting the influence of demographic and socio-economic factors on the T2DM burden (15). Sex-specific analysis further reveals nuanced differences in the contribution of dietary risks to T2DM across SDI levels. In low and middle SDI regions, men consistently exhibit higher contributions from dietary risks compared to women. Conversely, in high SDI regions, women show more significant reductions in T2DM burden due to dietary improvements, including reduced consumption of processed meat and sugar-sweetened beverages. This may reflect differences in health-seeking behaviors and the impact of gender-sensitive policies in high-income regions, which have facilitated better dietary and healthcare interventions for women. In low SDI regions, however, both men and women face compounded risks due to limited preventive care and significant dietary transitions, although cultural and social factors may uniquely influence these dynamics for each gender. These results underscore the need for dietary interventions and public health strategies tailored to regional and demographic contexts. Addressing the T2DM burden in elderly adults requires a multifaceted approach, with targeted policies focusing on low and middle SDI regions while maintaining dietary and healthcare improvements in high SDI regions. Additionally, incorporating sex-specific strategies can further enhance the effectiveness of interventions at a global scale.

Despite this study provides meaningful insights into the burden of T2DM attributable to dietary risk factors among elderly adults, several limitations should be acknowledged. First, the GBD 2021 framework does not account for the potential influence of the COVID-19 pandemic on T2DM prevalence and outcomes during 2020 and 2021 (45). This omission could bias estimates and obscure recent epidemiological trends. Second, the modeling approach used by GBD estimates risk based on available dietary data but does not establish causation, providing only approximations of the true burden. Insufficient data in many regions worldwide introduces uncertainties, particularly in low-income and underrepresented areas (46). To address this limitation, we analyzed regional-level data rather than focusing on individual countries. However, comparisons across countries should be interpreted cautiously due to inconsistencies in data sources, limited information on diabetes type, and variability in risk factors across income tiers and geographic locations. Additionally, the standardized evaluation framework used by the GBD is influenced by the diverse stages of economic development and the variable quality of healthcare systems globally. This limitation highlights the need for national and subnational diabetes registries to provide a more detailed understanding of diabetes risk factors and outcomes.

In recent years, public awareness of healthy eating remains insufficient, with persistent challenges such as imbalanced diets, excessive fat consumption, and inadequate dietary fiber intake. The increasing burden of T2DM among elderly adults attributable to dietary risks underscores the urgent need for public health interventions aimed at improving dietary quality. Leveraging data from the GBD 2021 study, this analysis provides a comprehensive overview of the burden of T2DM in elderly adults attributable to seven key dietary risk factors, offering a robust foundation for future research and targeted policy initiatives. This study underscores the significant impact of dietary factors on the T2DM burden in elderly populations, emphasizing the importance of addressing modifiable risks through strengthened public health measures. For elderly individuals, nutritional strategies should be personalized, taking into account their current dietary habits, willingness to adopt changes, and ability to access healthy food. These strategies must strike a balance between achieving metabolic control, maintaining adequate caloric intake, and ensuring nutritional quality. Nutritional therapy should be tailored to the broader context of the patient’s lifestyle, incorporating physical activity and medication regimens. Elderly diabetic patients are at higher risk of malnutrition, sarcopenia, and frailty compared to their non-diabetic peers (47). To mitigate these risks, it is essential to avoid overly restrictive energy intake while promoting balanced and adequate nutrition. Regular nutritional screenings are crucial for identifying and addressing malnutrition risks. However, traditional elderly care models, such as family-based and institutional care, tend to emphasize basic caregiving over comprehensive medical and healthcare support, thus failing to meet elderly adults’ individualized diabetes management needs adequately. To bridge this gap, novel care models integrating advanced healthcare services are necessary. Utilizing intelligent healthcare platforms offers a promising strategy: firstly, continuous monitoring of elderly individuals’ health indicators, such as glycemic control, nutritional intake, and lipid profiles, can be facilitated, allowing for the establishment of dynamic, personalized health records, using heart rate variability measured by a wearable device for early detection of hypoglycemia (48), utilizing wearable glucose biosensors for improved blood glucose monitoring (49), applying machine learning algorithms in combination with continuous glucose monitors to enhance nutritional interventions (50). Secondly, these platforms can rapidly capture diverse individual needs, enabling timely formulation of tailored nutritional plans. Such personalized strategies can be efficiently implemented through collaborative efforts among general practitioners, diabetes specialists, health managers, and community service workers. Regular nutritional screenings integrated within this model play an indispensable role in delivering tailored interventions, significantly enhancing the overall quality of elderly care. In addition, public health authorities could leverage community-based initiatives, such as nutrition awareness campaigns and cooking workshops, specifically designed for elderly care settings, including nursing homes and senior community centers. Policies could include incentives to healthcare providers and caregivers who implement successful nutritional education and screening programs. Furthermore, governments might consider implementing fiscal measures, such as subsidies for healthier foods and taxation of sugar-sweetened beverages and processed foods, to economically incentivize healthier dietary choices among elderly populations. By integrating policy guidance, educational outreach, and environmental improvements, this study provides actionable insights to inform strategies aimed at reducing the T2DM burden. Continued efforts to refine dietary interventions and address regional and socio-economic disparities will be essential for guiding populations toward healthier eating behaviors and mitigating the long-term impact of T2DM.

## 5 Conclusion

In conclusion, this study highlights the significant global burden of Type 2 Diabetes Mellitus (T2DM) attributable to dietary risks among elderly adults, emphasizing the critical influence of dietary patterns on health outcomes in this vulnerable population. The findings underscore the pressing need for targeted public health interventions and policies aimed at mitigating the impact of dietary risks on T2DM. Future research should prioritize exploring the intricate interplay between socio-economic disparities, aging, and dietary behaviors to inform the development of tailored strategies that address the specific needs of diverse regions and demographics. There is an urgent need to integrate precision nutrition approaches into healthcare systems, incorporating individual dietary habits, metabolic requirements, and socio-cultural contexts. Enhanced availability of longitudinal and sub-national data would improve modeling of dietary risk impacts, enabling more precise and actionable policy recommendations. Addressing the disproportionate burden in low and middle SDI regions will require international collaboration, with investments in health education, food system reforms, and healthcare infrastructure playing a pivotal role. Additionally, future research should investigate the influence of emerging dietary trends and the efficacy of novel interventions on T2DM prevention and management. These efforts will be crucial for developing global strategies to reduce the escalating public health challenge posed by T2DM, ensuring improved outcomes for aging populations worldwide.

## Data Availability

The datasets presented in this study can be found in online repositories. The names of the repository/repositories and accession number(s) can be found in the article/Supplementary material.

## Footnotes

^1^ https://vizhub.healthdata.org/gbd-results/

## Supplementary material

The Supplementary material for this article can befound online at: https://www.frontiersin.org/articles/10.3389/fnut.2025.1557923/full#supplementary-material
