# Supplementary material for: Global burden of Type 2 Diabetes Mellitus attributable to dietary risks in elderly adults: insights from the Global Burden of Disease study 2021
Source: Front Nutr. 2025 Apr 24;12:1557923. doi: 10.3389/fnut.2025.1557923 (PMC12058732; doi:10.3389/fnut.2025.1557923)
Supplement: Supplementary file 1 [file Data_Sheet_1.PDF]

Table of Contents

Supplement 1 Percent of T2DM burden in elderly adults attributable to dietary risk factors 1

Supplement 2 Age-standardized rates and rates changes attributable to dietary factors for T2DM burden in elderly adults from 1990 to 2021 2

Supplement 3 Decomposition of changes in the burden of T2DM attributable to dietary risk factors among elderly adults across SDI levels, 1990–2021.. 13

Supplement 1 Percent of T2DM burden in elderly adults attributable to dietary risk factors

Dietary Factors	1990		2021	
	Deaths Percent	DALYs Percent	Deaths Percent	DALYs Percent
Diet high in processed meat	0.0903(0.1477,0.0214)	0.0904(0.1525,0.0215)	0.072(0.1196,0.0168)	0.0825(0.1422,0.0194)
Diet high in red meat	0.0469(0.1049,-0.0068)	0.0486(0.1092,-0.0074)	0.0433(0.0971,-0.0061)	0.0496(0.1139,-0.0076)
Diet high in sugar-sweetened beverages	0.0278(0.0416,0.0139)	0.0276(0.0427,0.0137)	0.0314(0.0464,0.0158)	0.0354(0.0544,0.0179)
Diet low in fiber	0.014(0.02,0.0079)	0.0133(0.0193,0.0074)	0.0111(0.0161,0.0062)	0.0104(0.0155,0.0057)
Diet low in fruits	0.0495(0.0883,0.0079)	0.0501(0.0904,0.0078)	0.0464(0.0818,0.0071)	0.0418(0.0757,0.0064)
Diet low in vegetables	0.0178(0.0379,-0.0066)	0.0156(0.0335,-0.0059)	0.0097(0.0215,-0.0036)	0.0076(0.0168,-0.0029)
Diet low in whole grains	0.0742(0.1208,0.0215)	0.0749(0.1265,0.0218)	0.0651(0.1066,0.0182)	0.0671(0.1144,0.0189)
Dietary risks	0.2653 (0.4298,0.05)	0.2658(0.4416,0.051)	0.2361(0.3873,0.0453)	0.2485(0.4181,0.0522)

Supplement 2 Age-standardized rates and rates changes attributable to dietary factors for T2DM burden in elderly adults from 1990 to 2021

Location	Dietary Factors	Age-Standardized Rate Per 100,000 People (95 % UI)				Average Annual Percent Change from 1990 to 2021(95% CI)	
		1990		2021			
		Death Rate	DALYs Rate	Death Rate	DALYs Rate	Death Rate	DALYs Rate
Global	Diet high in processed meat	12.92 (3.06-21.17)	302.67 (71.95-510.06)	11.09 (2.59-18.43)	362.95 (85.28-625.45)	-0.47 (-0.69 to -0.25)	0.59 (0.51 to 0.66)
	Diet high in red meat	6.59 (-0.95-14.74)	160.24 (-24.17-360.88)	6.62 (-0.92-14.86)	216.99 (-33.11-498.27)	0.02 (-0.17 to 0.22)	0.99 (0.88 to 1.1)
	Diet high in sugar-sweetened beverages	3.94 (1.97-5.94)	91.87 (45.71-142.35)	4.80 (2.41-7.11)	154.98 (78.22-238.14)	0.65 (0.46 to 0.83)	1.72 (1.67 to 1.76)
	Diet low in fiber	1.99 (1.12-2.85)	44.72 (25.03-64.87)	1.71 (0.96-2.49)	46.23 (25.45-68.54)	-0.46 (-0.63 to -0.29)	0.11 (0.01 to 0.22)
	Diet low in fruits	6.78 (1.09-12.11)	164.08 (25.63-296.03)	7.07 (1.09-12.46)	183.64 (28.33-332.18)	0.15 (-0.06 to 0.36)	0.36 (0.25 to 0.47)
	Diet low in vegetables	2.44 (-0.91-5.22)	51.69 (-19.40-110.64)	1.49 (-0.56-3.30)	33.57 (-12.70-74.45)	-1.56 (-1.64 to -1.48)	-1.38 (-1.43 to -1.33)
	Diet low in whole grains	10.41 (3.02-17.01)	248.45 (72.14-419.18)	9.98 (2.79-16.36)	295.04 (83.09-502.57)	-0.12 (-0.25 to 0.01)	0.56 (0.51 to 0.61)
	Dietary risks	37.27 (7.09-60.39)	881.16 (170.00-1461.83)	36.16 (6.95-59.35)	1091.50 (229.37-1835.49)	-0.08 (-0.22 to 0.06)	0.7 (0.63 to 0.77)
High SDI	Diet high in processed meat	19.10 (4.56-31.03)	470.88 (113.21-779.56)	12.58 (3.04-20.48)	593.50 (139.68-1026.79)	-1.32 (-1.56 to -1.09)	0.75 (0.67 to 0.83)
	Diet high in red meat	8.86 (-1.28-19.60)	219.88 (-33.26-488.37)	6.14 (-0.89-13.63)	272.03 (-42.81-627.41)	-1.15 (-1.41 to -0.89)	0.71 (0.53 to 0.88)
	Diet high in sugar-sweetened beverages	5.34 (2.52-8.48)	133.77 (63.30-215.27)	4.64 (2.27-7.15)	222.92 (109.09-365.39)	-0.48 (-0.67 to -0.29)	1.68 (1.5 to 1.86)
	Diet low in fiber	1.53 (0.86-2.21)	34.75 (19.25-50.69)	0.85 (0.46-1.24)	34.70 (18.60-54.65)	-1.89 (-2.09 to -1.68)	-0.02 (-0.09 to 0.06)
	Diet low in fruits	3.68 (0.60-6.67)	92.78 (14.85-171.09)	1.91 (0.31-3.56)	85.07 (13.89-164.93)	-2.1 (-2.35 to -1.86)	-0.27 (-0.39 to -0.16)
	Diet low in vegetables	0.29 (-0.10-0.64)	6.52 (-2.32-14.74)	0.17 (-0.06-0.40)	6.01 (-2.09-14.62)	-1.72 (-2.08 to -1.36)	-0.26 (-0.42 to -0.1)
	Diet low in whole grains	6.81 (1.93-11.42)	164.30 (46.56-281.84)	5.12 (1.44-8.69)	214.20 (59.20-383.02)	-0.93 (-1.16 to -0.69)	0.87 (0.76 to 0.98)
	Dietary risks	37.15 (7.65-59.02)	916.34 (197.40-1482.24)	25.59 (5.64-40.49)	1165.13 (274.40-1946.14)	-1.18 (-1.44 to -0.93)	0.78 (0.64 to 0.93)
High-Middle SDI	Diet high in processed meat	12.33 (2.91-20.16)	311.05 (73.82-527.07)	11.61 (2.72-19.09)	354.74 (83.79-612.90)	-0.14 (-0.43 to 0.15)	0.44 (0.3 to 0.57)
	Diet high in red meat	7.05 (-1.03-15.73)	182.39 (-28.06-412.66)	7.20 (-1.06-16.41)	236.00 (-38.34-539.49)	0.1 (-0.16 to 0.36)	0.82 (0.68 to 0.96)

	Diet high in sugar-sweetened beverages	3.65 (1.80-5.77)	84.92 (41.81-133.09)	4.04 (1.99-6.15)	128.17 (62.78-201.79)	0.38 (0.15 to 0.61)	1.36 (1.27 to 1.46)
	Diet low in fiber	1.09 (0.59-1.62)	26.54 (14.37-40.22)	0.87 (0.47-1.33)	25.43 (13.31-39.84)	-0.69 (-0.95 to -0.43)	-0.14 (-0.3 to 0.03)
	Diet low in fruits	2.95 (0.46-5.34)	89.34 (13.65-164.95)	2.34 (0.38-4.38)	68.85 (11.12-130.81)	-0.71 (-1.04 to -0.39)	-0.85 (-0.97 to -0.73)
	Diet low in vegetables	0.21 (-0.07-0.49)	5.34 (-1.89-12.27)	0.10 (-0.03-0.23)	2.73 (-0.93-6.42)	-2.4 (-2.77 to -2.04)	-2.14 (-2.48 to -1.8)
	Diet low in whole grains	14.48 (4.23-23.53)	351.75 (102.25-585.24)	12.15 (3.46-19.85)	357.74 (102.71-606.47)	-0.54 (-0.77 to -0.31)	0.06 (-0.07 to 0.19)
	Dietary risks	34.24 (7.48-53.79)	861.46 (193.29-1396.21)	32.07 (6.78-51.74)	983.50 (216.95-1637.86)	-0.19 (-0.44 to 0.06)	0.44 (0.31 to 0.56)
Middle SDI	Diet high in processed meat	4.99 (1.15-8.73)	116.56 (26.23-204.46)	6.64 (1.52-11.58)	183.40 (42.29-326.26)	0.97 (0.78 to 1.17)	1.49 (1.35 to 1.63)
	Diet high in red meat	5.31 (-0.76-12.23)	129.90 (-19.42-303.59)	7.90 (-1.07-18.10)	225.77 (-33.11-518.26)	1.33 (0.87 to 1.8)	1.82 (1.58 to 2.07)
	Diet high in sugar-sweetened beverages	3.61 (1.75-5.87)	80.00 (38.33-130.07)	5.90 (2.86-9.19)	153.85 (75.44-242.17)	1.63 (1.18 to 2.09)	2.19 (1.94 to 2.45)
	Diet low in fiber	2.51 (1.41-3.63)	56.94 (31.40-83.10)	2.03 (1.11-2.98)	51.48 (27.91-76.52)	-0.7 (-0.8 to -0.6)	-0.35 (-0.42 to -0.27)
	Diet low in fruits	8.62 (1.36-15.28)	203.44 (31.39-366.80)	7.91 (1.20-14.10)	189.39 (29.24-340.99)	-0.26 (-0.45 to -0.08)	-0.23 (-0.33 to -0.12)
	Diet low in vegetables	2.03 (-0.74-4.59)	42.57 (-15.97-96.65)	0.66 (-0.24-1.52)	15.46 (-5.56-35.82)	-3.56 (-3.77 to -3.35)	-3.26 (-3.42 to -3.1)
	Diet low in whole grains	7.96 (2.23-13.26)	198.31 (57.02-338.57)	8.94 (2.47-15.01)	256.26 (71.04-438.05)	0.39 (0.17 to 0.61)	0.82 (0.72 to 0.93)
	Dietary risks	29.89 (5.01-51.05)	707.59 (118.34-1233.62)	34.77 (5.98-59.55)	935.72 (169.85-1629.03)	0.53 (0.33 to 0.74)	0.91 (0.77 to 1.05)
Low-middle SDI	Diet high in processed meat	8.63 (2.03-14.95)	180.08 (41.72-314.35)	13.29 (3.05-23.02)	306.01 (70.73-531.11)	1.42 (1.06 to 1.78)	1.75 (1.59 to 1.91)
	Diet high in red meat	2.53 (-0.34-5.94)	56.17 (-7.79-131.72)	4.55 (-0.63-10.56)	113.34 (-16.27-263.60)	1.93 (1.73 to 2.13)	2.3 (2.14 to 2.46)
	Diet high in sugar-sweetened beverages	1.81 (0.91-2.83)	41.22 (20.53-64.24)	4.70 (2.30-7.23)	113.08 (55.08-174.95)	3.2 (2.83 to 3.57)	3.32 (3.14 to 3.49)
	Diet low in fiber	4.00 (2.28-5.76)	81.93 (46.67-118.52)	4.25 (2.36-6.21)	90.80 (50.37-133.56)	0.23 (0.07 to 0.4)	0.36 (0.13 to 0.58)
	Diet low in fruits	15.66 (2.50-28.21)	339.46 (52.47-609.36)	20.70 (3.10-36.20)	461.54 (67.82-812.47)	0.99 (0.6 to 1.38)	1.06 (0.9 to 1.21)
	Diet low in vegetables	8.10 (-2.89-17.53)	157.02 (-57.50-340.15)	4.86 (-1.75-11.04)	90.86 (-34.05-210.32)	-1.56 (-2.07 to -1.04)	-1.73 (-2.04 to -1.42)

	Diet low in whole grains	12.67 (3.73-21.09)	279.49 (81.79-470.81)	15.40 (4.19-25.75)	372.95 (103.41-627.47)	0.68 (0.37 to 0.98)	0.94 (0.79 to 1.1)
	Dietary risks	44.13 (7.11-74.97)	943.50 (159.46-1618.34)	57.68 (11.09-97.01)	1325.63 (270.48-2229.20)	0.88 (0.83 to 0.93)	1.12 (0.9 to 1.33)
Low SDI	Diet high in processed meat	14.40 (3.39-24.82)	298.54 (69.17-515.51)	16.97 (3.93-29.08)	381.21 (87.28-660.51)	0.56 (0.49 to 0.62)	0.8 (0.74 to 0.86)
	Diet high in red meat	2.80 (-0.38-6.63)	60.61 (-8.69-142.23)	3.28 (-0.44-7.75)	76.78 (-10.94-185.41)	0.53 (0.47 to 0.6)	0.78 (0.72 to 0.83)
	Diet high in sugar-sweetened beverages	1.72 (0.83-2.78)	35.83 (17.30-57.85)	2.41 (1.17-3.79)	53.98 (25.90-85.16)	1.14 (1.04 to 1.24)	1.36 (1.31 to 1.42)
	Diet low in fiber	2.48 (1.39-3.68)	50.00 (27.62-73.70)	2.54 (1.40-3.90)	55.43 (30.09-83.33)	0.08 (-0.23 to 0.4)	0.34 (0.21 to 0.47)
	Diet low in fruits	18.20 (2.86-32.53)	378.60 (57.85-677.88)	20.65 (3.10-36.45)	460.44 (68.25-819.24)	0.45 (0.29 to 0.61)	0.66 (0.5 to 0.82)
	Diet low in vegetables	19.36 (-7.44-41.36)	386.27 (-149.22-821.02)	14.97 (-5.59-32.62)	314.74 (-119.89-686.10)	-0.8 (-0.93 to -0.67)	-0.66 (-0.73 to -0.6)
	Diet low in whole grains	20.23 (5.89-33.64)	423.81 (122.04-711.50)	19.95 (5.67-33.24)	451.37 (125.52-755.00)	-0.01 (-0.14 to 0.12)	0.22 (0.12 to 0.33)
	Dietary risks	67.07 (8.99-113.83)	1383.30 (190.46-2349.81)	69.28 (10.53-116.72)	1538.55 (247.19-2615.87)	0.13 (0 to 0.26)	0.36 (0.27 to 0.45)
Andean Latin America	Diet high in processed meat	2.70 (0.58-4.86)	66.31 (13.97-119.93)	4.04 (0.85-7.37)	121.95 (25.22-222.96)	1.3 (0.8 to 1.8)	2.02 (1.63 to 2.4)
	Diet high in red meat	4.67 (-0.65-11.07)	112.02 (-16.41-265.71)	7.43 (-1.01-17.80)	219.67 (-30.90-534.19)	1.56 (1.14 to 1.99)	2.2 (1.91 to 2.5)
	Diet high in sugar-sweetened beverages	4.22 (1.73-7.56)	104.74 (42.69-188.08)	8.14 (3.52-14.74)	244.10 (105.65-430.40)	2.11 (1.63 to 2.59)	2.78 (2.54 to 3.02)
	Diet low in fiber	2.47 (1.35-3.66)	57.45 (31.73-85.19)	2.61 (1.36-4.04)	72.59 (37.88-113.29)	0.18 (-0.45 to 0.82)	0.74 (0.31 to 1.16)
	Diet low in fruits	4.03 (0.58-7.77)	93.56 (13.34-174.63)	3.27 (0.50-7.05)	83.52 (12.23-177.67)	-0.63 (-1.56 to 0.32)	-0.25 (-0.64 to 0.14)
	Diet low in vegetables	4.48 (-1.53-10.38)	101.43 (-35.40-236.33)	5.44 (-1.87-13.40)	151.71 (-52.75-368.35)	0.53 (-0.32 to 1.39)	1.22 (0.71 to 1.73)
	Diet low in whole grains	11.66 (3.09-19.92)	277.46 (71.77-479.67)	10.04 (2.65-17.94)	280.62 (72.62-500.03)	-0.43 (-0.92 to 0.05)	0.04 (-0.23 to 0.31)
	Dietary risks	28.96 (5.00-50.88)	688.65 (120.40-1219.39)	34.89 (5.75-63.05)	999.05 (161.19-1808.37)	0.65 (0.1 to 1.22)	1.22 (0.9 to 1.54)
Australasia	Diet high in processed meat	17.83 (4.29-29.82)	424.05 (99.69-719.86)	14.22 (3.45-23.73)	482.62 (115.41-848.15)	-0.69 (-1.48 to 0.1)	0.44 (0.02 to 0.86)
	Diet high in red meat	11.73 (-2.02-25.59)	281.84 (-53.09-622.58)	8.81 (-1.49-19.08)	300.42 (-59.22-679.71)	-0.89 (-1.68 to -0.09)	0.22 (-0.12 to 0.57)

	Diet high in sugar-sweetened beverages	6.15 (2.57-11.28)	144.94 (61.10-262.53)	4.96 (2.06-8.88)	170.67 (72.47-302.93)	-0.65 (-1.36 to 0.07)	0.54 (0.23 to 0.85)
	Diet low in fiber	1.47 (0.78-2.26)	33.45 (18.13-50.37)	0.87 (0.42-1.42)	31.60 (15.58-52.24)	-1.66 (-2.4 to -0.91)	-0.17 (-0.39 to 0.05)
	Diet low in fruits	3.95 (0.59-7.61)	89.10 (12.65-170.78)	2.38 (0.32-4.93)	74.25 (10.07-154.10)	-1.58 (-2.27 to -0.88)	-0.58 (-0.98 to -0.17)
	Diet low in vegetables	0.17 (-0.05-0.40)	3.93 (-1.25-9.32)	0.12 (-0.04-0.29)	3.94 (-1.30-9.73)	-1.01 (-1.8 to -0.21)	0.03 (-0.33 to 0.39)
	Diet low in whole grains	3.54 (0.93-6.69)	81.54 (21.16-149.73)	3.75 (0.95-6.92)	127.71 (32.29-236.26)	0.23 (-0.45 to 0.92)	1.48 (1.12 to 1.84)
	Dietary risks	36.17 (6.57-59.38)	854.28 (152.79-1423.83)	28.42 (5.52-46.01)	957.48 (190.11-1613.18)	-0.74 (-1.54 to 0.06)	0.39 (0.04 to 0.73)
Caribbean	Diet high in processed meat	13.08 (2.98-23.01)	303.00 (67.27-538.58)	11.25 (2.61-19.95)	344.88 (76.51-624.44)	-0.47 (-0.9 to -0.04)	0.42 (0.15 to 0.7)
	Diet high in red meat	8.10 (-1.10-19.44)	189.90 (-26.28-459.90)	6.61 (-0.81-16.09)	215.47 (-28.81-533.26)	-0.65 (-1.03 to -0.27)	0.38 (0.18 to 0.57)
	Diet high in sugar-sweetened beverages	8.06 (3.59-13.53)	180.46 (78.45-307.03)	7.64 (3.42-12.72)	234.78 (105.93-406.29)	-0.2 (-0.71 to 0.31)	0.84 (0.47 to 1.2)
	Diet low in fiber	4.53 (2.52-6.60)	99.14 (54.66-145.75)	2.88 (1.54-4.43)	76.19 (40.19-117.14)	-1.43 (-1.7 to -1.17)	-0.85 (-1.05 to -0.65)
	Diet low in fruits	8.48 (1.26-15.67)	182.52 (27.39-342.89)	5.80 (0.87-11.36)	149.38 (22.15-294.29)	-1.17 (-1.39 to -0.95)	-0.64 (-0.77 to -0.5)
	Diet low in vegetables	13.27 (-4.72-29.06)	279.59 (-102.01-618.01)	9.88 (-3.67-23.46)	243.20 (-93.23-566.66)	-0.94 (-1.08 to -0.81)	-0.43 (-0.61 to -0.25)
	Diet low in whole grains	31.51 (8.77-52.51)	696.67 (187.76-1171.72)	22.46 (6.01-38.43)	612.07 (165.70-1059.14)	-1.06 (-1.42 to -0.7)	-0.41 (-0.63 to -0.2)
	Dietary risks	74.15 (13.64-125.41)	1647.52 (302.88-2819.21)	57.47 (10.25-101.07)	1628.99 (305.35-2916.73)	-0.79 (-1.11 to -0.47)	-0.03 (-0.21 to 0.14)
Central Asia	Diet high in processed meat	8.13 (1.96-13.45)	269.28 (62.64-473.02)	16.73 (3.84-28.81)	580.03 (133.15-1022.30)	2.35 (2.01 to 2.69)	2.49 (2.2 to 2.78)
	Diet high in red meat	3.37 (-0.48-7.39)	117.99 (-18.20-278.92)	7.55 (-1.05-17.50)	271.68 (-40.37-635.59)	2.66 (2.27 to 3.06)	2.73 (2.38 to 3.09)
	Diet high in sugar-sweetened beverages	1.22 (0.52-2.22)	42.93 (17.90-82.63)	3.30 (1.48-5.85)	118.96 (53.30-212.29)	3.29 (2.79 to 3.78)	3.38 (2.98 to 3.78)
	Diet low in fiber	0.77 (0.44-1.12)	24.46 (13.66-36.54)	1.08 (0.58-1.65)	35.84 (18.68-55.86)	1.07 (0.45 to 1.69)	1.26 (0.93 to 1.59)
	Diet low in fruits	3.02 (0.45-5.30)	102.10 (14.68-190.39)	3.35 (0.50-6.27)	116.78 (16.31-224.56)	0.28 (-0.32 to 0.89)	0.39 (0.06 to 0.72)
	Diet low in vegetables	0.15 (-0.06-0.35)	5.19 (-1.87-11.69)	0.05 (-0.02-0.13)	1.68 (-0.53-4.34)	-3.56 (-4.02 to -3.09)	-3.63 (-4.07 to -3.17)

	Diet low in whole grains	11.87 (3.50-18.69)	383.20 (110.97-637.36)	23.54 (6.58-38.50)	797.46 (220.79-1333.10)	2.23 (1.89 to 2.58)	2.41 (2.24 to 2.57)
	Dietary risks	22.90 (5.34-34.60)	753.35 (185.43-1210.82)	45.27 (10.81-71.55)	1557.67 (392.06-2526.88)	2.23 (1.91 to 2.55)	2.38 (2.17 to 2.59)
Central Europe	Diet high in processed meat	13.14 (3.17-21.72)	420.02 (96.52-727.65)	17.70 (4.23-29.12)	627.82 (148.53-1095.85)	0.94 (0.6 to 1.29)	1.28 (1.13 to 1.44)
	Diet high in red meat	6.97 (-1.02-15.62)	229.13 (-35.69-529.76)	8.70 (-1.25-19.55)	317.06 (-51.15-736.62)	0.67 (0.25 to 1.09)	1.04 (0.87 to 1.21)
	Diet high in sugar-sweetened beverages	4.87 (2.34-7.49)	155.82 (74.67-247.96)	7.53 (3.65-11.73)	273.22 (131.43-439.01)	1.38 (0.95 to 1.81)	1.81 (1.64 to 1.98)
	Diet low in fiber	0.96 (0.54-1.40)	28.70 (15.78-43.15)	1.12 (0.61-1.68)	35.38 (18.77-55.01)	0.48 (0.01 to 0.95)	0.63 (0.38 to 0.89)
	Diet low in fruits	3.78 (0.58-6.76)	120.51 (18.22-223.66)	3.63 (0.60-6.69)	121.52 (18.86-235.37)	-0.14 (-0.79 to 0.51)	0 (-0.22 to 0.21)
	Diet low in vegetables	0.13 (-0.04-0.30)	4.01 (-1.32-9.49)	0.12 (-0.04-0.30)	3.79 (-1.27-9.35)	-0.14 (-0.63 to 0.36)	-0.19 (-0.46 to 0.09)
	Diet low in whole grains	11.58 (3.26-18.91)	375.42 (103.96-635.76)	10.32 (2.82-17.11)	344.91 (95.31-607.40)	-0.39 (-0.82 to 0.05)	-0.3 (-0.53 to -0.08)
	Dietary risks	33.98 (7.42-53.33)	1091.97 (254.22-1794.37)	40.58 (9.30-64.27)	1417.37 (331.93-2376.68)	0.54 (0.16 to 0.92)	0.82 (0.65 to 0.99)
Central Latin America	Diet high in processed meat	17.70 (4.03-32.84)	412.04 (92.15-764.42)	18.95 (4.40-34.80)	482.02 (110.41-889.66)	0.31 (-0.38 to 0.99)	0.53 (0.1 to 0.96)
	Diet high in red meat	18.72 (-2.66-43.46)	435.18 (-62.69-998.27)	19.54 (-2.61-46.66)	486.64 (-67.93-1149.59)	0.22 (-0.49 to 0.93)	0.39 (-0.01 to 0.78)
	Diet high in sugar-sweetened beverages	19.60 (7.45-37.18)	447.78 (171.60-844.65)	21.15 (8.89-38.47)	522.99 (223.30-937.99)	0.33 (-0.27 to 0.93)	0.51 (0.11 to 0.91)
	Diet low in fiber	1.91 (1.03-2.94)	43.22 (23.26-65.56)	2.05 (1.04-3.25)	51.46 (26.23-81.45)	0.24 (-0.3 to 0.77)	0.53 (0.21 to 0.85)
	Diet low in fruits	6.81 (1.12-13.10)	151.99 (24.65-288.08)	5.99 (0.91-11.99)	143.59 (21.76-283.44)	-0.43 (-0.94 to 0.08)	-0.24 (-0.55 to 0.08)
	Diet low in vegetables	2.94 (-1.04-6.94)	67.00 (-24.57-155.57)	1.51 (-0.51-3.61)	37.42 (-13.29-88.74)	-2.17 (-2.59 to -1.74)	-1.92 (-2.21 to -1.63)
	Diet low in whole grains	7.36 (1.95-13.12)	170.85 (45.53-302.94)	7.72 (2.05-14.21)	196.45 (51.95-356.22)	0.18 (-0.3 to 0.66)	0.43 (0.15 to 0.71)
	Dietary risks	66.90 (13.56-115.06)	1539.47 (299.71-2681.61)	68.42 (13.35-119.99)	1705.14 (332.10-3017.93)	0.13 (-0.51 to 0.77)	0.33 (-0.02 to 0.68)
Central Sub-Saharan Africa	Diet high in processed meat	21.53 (4.91-42.82)	435.52 (100.14-853.04)	20.76 (4.23-40.58)	458.82 (93.62-880.75)	-0.12 (-0.17 to -0.07)	0.15 (0.09 to 0.21)
	Diet high in red meat	4.96 (-0.77-11.64)	97.67 (-15.40-226.67)	4.87 (-0.66-11.81)	110.71 (-15.59-268.00)	-0.05 (-0.16 to 0.05)	0.4 (0.3 to 0.5)

	Diet high in sugar-sweetened beverages	4.60 (1.63-10.09)	91.64 (33.17-199.89)	3.92 (1.58-7.77)	85.17 (34.04-169.28)	-0.5 (-0.59 to -0.41)	-0.24 (-0.39 to -0.09)
	Diet low in fiber	2.97 (1.57-4.61)	55.49 (29.74-85.07)	4.14 (1.99-6.90)	83.29 (40.72-135.43)	1.07 (0.93 to 1.22)	1.31 (1.17 to 1.46)
	Diet low in fruits	16.48 (2.50-30.93)	318.36 (48.08-585.73)	23.30 (3.19-44.42)	489.51 (66.69-929.45)	1.13 (1.02 to 1.24)	1.4 (1.31 to 1.48)
	Diet low in vegetables	50.96 (-18.34-110.69)	995.79 (-364.49-2156.55)	45.22 (-15.37-101.23)	932.38 (-326.57-2067.47)	-0.37 (-0.44 to -0.3)	-0.2 (-0.28 to -0.12)
	Diet low in whole grains	14.76 (3.99-26.10)	289.70 (78.23-502.35)	16.38 (4.11-30.05)	347.76 (86.44-620.95)	0.36 (0.27 to 0.45)	0.58 (0.49 to 0.68)
	Dietary risks	100.31 (1.87-185.89)	1970.73 (46.96-3614.70)	101.24 (4.01-189.71)	2142.83 (94.87-3931.35)	0.03 (-0.09 to 0.15)	0.27 (0.16 to 0.38)
East Asia	Diet high in processed meat	1.88 (0.40-3.36)	52.02 (11.02-94.10)	3.10 (0.69-5.76)	107.18 (24.37-203.97)	1.67 (1.35 to 1.99)	2.33 (2 to 2.67)
	Diet high in red meat	3.63 (-0.52-9.00)	104.04 (-15.64-258.36)	6.32 (-0.92-15.05)	214.07 (-33.07-496.76)	1.9 (1.55 to 2.24)	2.35 (2.14 to 2.55)
	Diet high in sugar-sweetened beverages	0.41 (0.17-0.78)	10.99 (4.59-20.35)	1.31 (0.54-2.51)	43.81 (18.34-81.12)	3.89 (3.65 to 4.14)	4.58 (4.32 to 4.85)
	Diet low in fiber	1.00 (0.48-1.66)	26.41 (12.31-44.94)	0.54 (0.22-1.00)	15.98 (6.59-30.67)	-2.03 (-2.3 to -1.76)	-1.65 (-1.91 to -1.38)
	Diet low in fruits	4.81 (0.73-8.87)	137.92 (20.90-256.74)	1.94 (0.28-4.11)	56.48 (7.92-119.89)	-2.87 (-3.23 to -2.51)	-2.83 (-3.06 to -2.61)
	Diet low in vegetables	0.13 (-0.04-0.31)	3.82 (-1.27-8.84)	0.03 (-0.01-0.08)	0.68 (-0.19-2.10)	-5.02 (-5.31 to -4.72)	-5.46 (-5.63 to -5.29)
	Diet low in whole grains	5.22 (1.37-9.09)	146.78 (40.50-258.80)	5.69 (1.57-10.41)	183.80 (51.04-332.88)	0.31 (0 to 0.62)	0.73 (0.49 to 0.97)
	Dietary risks	14.76 (1.81-26.09)	418.25 (53.36-746.44)	16.69 (1.93-30.70)	548.44 (64.66-1001.33)	0.43 (0.15 to 0.72)	0.87 (0.64 to 1.11)
Eastern Europe	Diet high in processed meat	5.29 (1.28-8.49)	245.31 (58.97-416.00)	21.27 (5.02-34.30)	616.68 (149.16-1023.60)	4.72 (4.1 to 5.35)	3.04 (2.76 to 3.32)
	Diet high in red meat	2.23 (-0.33-4.91)	105.08 (-16.34-238.72)	6.71 (-0.91-15.75)	201.04 (-28.36-478.94)	3.81 (2.94 to 4.68)	2.13 (1.85 to 2.42)
	Diet high in sugar-sweetened beverages	0.48 (0.21-0.85)	22.45 (9.56-41.12)	2.88 (1.16-5.53)	85.26 (35.35-157.61)	6.17 (5.57 to 6.77)	4.39 (3.86 to 4.92)
	Diet low in fiber	0.23 (0.13-0.33)	10.36 (5.49-15.94)	1.06 (0.53-1.75)	29.32 (14.97-47.79)	5.13 (4.02 to 6.26)	3.47 (3.01 to 3.93)
	Diet low in fruits	1.63 (0.25-2.93)	76.38 (11.82-141.54)	4.70 (0.78-8.87)	139.99 (23.02-266.54)	3.58 (2.71 to 4.46)	1.99 (1.81 to 2.17)
	Diet low in vegetables	0.03 (-0.01-0.08)	1.61 (-0.53-3.83)	0.15 (-0.05-0.36)	4.42 (-1.49-10.34)	5.03 (4.17 to 5.9)	3.35 (3.08 to 3.62)

	Diet low in whole grains	4.23 (1.19-6.88)	199.98 (56.95-340.01)	11.77 (3.31-19.68)	356.09 (98.79-608.21)	3.44 (2.62 to 4.27)	1.93 (1.66 to 2.2)
	Dietary risks	11.17 (2.32-17.01)	523.67 (116.26-844.44)	39.70 (9.05-61.34)	1173.83 (272.25-1877.83)	4.26 (3.4 to 5.13)	2.66 (2.42 to 2.9)
Eastern Sub-Saharan Africa	Diet high in processed meat	17.15 (4.01-30.66)	335.92 (78.54-598.96)	17.85 (4.23-31.55)	369.63 (86.23-648.64)	0.15 (0.05 to 0.25)	0.31 (0.25 to 0.37)
	Diet high in red meat	4.08 (-0.55-9.72)	79.45 (-10.85-187.49)	4.35 (-0.56-10.53)	90.43 (-12.17-215.78)	0.23 (0.17 to 0.28)	0.42 (0.38 to 0.46)
	Diet high in sugar-sweetened beverages	2.72 (1.27-4.49)	51.90 (24.14-85.20)	3.82 (1.81-6.21)	77.59 (37.09-126.96)	1.13 (1 to 1.26)	1.33 (1.24 to 1.41)
	Diet low in fiber	2.06 (1.11-3.07)	37.68 (20.40-55.71)	1.48 (0.77-2.34)	28.65 (14.96-44.61)	-1.06 (-1.14 to -0.98)	-0.9 (-1.01 to -0.79)
	Diet low in fruits	24.19 (3.78-42.71)	471.29 (72.21-835.80)	20.73 (3.39-36.37)	415.06 (65.50-735.21)	-0.47 (-0.59 to -0.36)	-0.4 (-0.46 to -0.34)
	Diet low in vegetables	34.22 (-13.32-73.00)	651.07 (-252.51-1383.37)	22.08 (-8.12-48.39)	419.69 (-159.20-922.63)	-1.42 (-1.53 to -1.32)	-1.43 (-1.54 to -1.31)
	Diet low in whole grains	22.84 (6.40-38.32)	449.82 (123.96-751.68)	18.46 (5.18-31.36)	366.81 (102.86-620.93)	-0.69 (-0.79 to -0.59)	-0.66 (-0.76 to -0.57)
	Dietary risks	91.23 (9.20-157.56)	1763.64 (181.84-3048.81)	77.05 (9.61-132.92)	1534.48 (202.18-2643.37)	-0.54 (-0.62 to -0.45)	-0.46 (-0.53 to -0.39)
High-income Asia Pacific	Diet high in processed meat	9.74 (2.33-16.60)	357.67 (85.30-620.95)	4.11 (0.97-7.01)	438.08 (100.33-812.69)	-2.8 (-3.16 to -2.44)	0.67 (0.48 to 0.86)
	Diet high in red meat	2.26 (-0.31-5.58)	76.09 (-10.58-184.06)	1.47 (-0.21-3.55)	126.72 (-18.60-318.77)	-1.44 (-2 to -0.88)	1.65 (1.5 to 1.81)
	Diet high in sugar-sweetened beverages	2.23 (0.91-4.21)	74.75 (30.98-138.31)	1.04 (0.45-1.90)	104.47 (44.57-192.93)	-2.46 (-2.89 to -2.03)	1.13 (0.82 to 1.45)
	Diet low in fiber	0.89 (0.47-1.34)	25.70 (13.58-39.69)	0.57 (0.30-0.88)	36.64 (18.98-59.12)	-1.46 (-1.96 to -0.95)	1.12 (0.84 to 1.41)
	Diet low in fruits	3.52 (0.56-6.34)	112.27 (18.11-208.48)	1.07 (0.17-2.11)	86.34 (13.59-178.39)	-3.81 (-4.2 to -3.41)	-0.85 (-1.07 to -0.64)
	Diet low in vegetables	0.08 (-0.03-0.19)	2.48 (-0.81-6.05)	0.03 (-0.01-0.07)	1.96 (-0.65-5.33)	-3.06 (-3.63 to -2.49)	-0.78 (-0.99 to -0.56)
	Diet low in whole grains	1.63 (0.42-3.02)	52.17 (13.67-94.84)	1.06 (0.28-1.96)	105.85 (28.65-201.51)	-1.39 (-1.84 to -0.94)	2.34 (2.01 to 2.66)
	Dietary risks	17.54 (3.93-29.21)	606.56 (142.03-1028.14)	7.87 (1.62-13.19)	765.42 (172.60-1365.85)	-2.61 (-2.98 to -2.25)	0.76 (0.59 to 0.93)
High-income North America	Diet high in processed meat	22.49 (5.30-36.41)	595.39 (142.01-979.95)	18.46 (4.52-29.70)	937.29 (223.93-1593.67)	-0.63 (-1.01 to -0.25)	1.45 (1.34 to 1.57)
	Diet high in red meat	9.99 (-1.49-22.21)	274.62 (-42.32-603.92)	7.85 (-1.16-17.67)	408.23 (-64.33-950.57)	-0.77 (-1.15 to -0.38)	1.26 (1.14 to 1.39)

	Diet high in sugar-sweetened beverages	6.85 (2.75-12.44)	188.15 (77.48-340.90)	7.38 (3.19-12.52)	396.81 (173.16-687.18)	0.23 (-0.14 to 0.59)	2.43 (2.21 to 2.65)
	Diet low in fiber	1.83 (1.02-2.68)	44.07 (24.13-64.76)	0.95 (0.47-1.55)	43.58 (21.12-72.81)	-2.1 (-2.38 to -1.83)	-0.06 (-0.24 to 0.11)
	Diet low in fruits	3.66 (0.58-6.84)	92.02 (14.42-172.26)	2.14 (0.31-4.47)	103.90 (14.88-219.54)	-1.7 (-1.99 to -1.42)	0.43 (0.35 to 0.51)
	Diet low in vegetables	0.16 (-0.05-0.39)	4.09 (-1.33-9.54)	0.18 (-0.06-0.48)	8.74 (-2.94-22.95)	0.35 (0.04 to 0.67)	2.49 (2.25 to 2.73)
	Diet low in whole grains	6.37 (1.78-11.00)	158.21 (43.58-275.04)	5.33 (1.47-9.64)	263.64 (70.45-487.19)	-0.56 (-0.92 to -0.2)	1.68 (1.45 to 1.92)
	Dietary risks	41.54 (9.14-65.06)	1096.45 (247.18-1758.66)	34.23 (8.42-53.15)	1744.93 (440.02-2872.35)	-0.62 (-0.99 to -0.25)	1.49 (1.38 to 1.61)
North Africa and Middle Eas	Diet high in processed meat	5.15 (1.14-9.06)	120.32 (26.63-214.57)	120.32 (26.63-214.57)	246.92 (55.77-445.59)	1.54 (1.3 to 1.77)	2.35 (2.23 to 2.48)
	Diet high in red meat	3.79 (-0.50-9.07)	91.48 (-12.77-218.01)	91.48 (-12.77-218.01)	156.39 (-21.91-381.40)	1.02 (0.78 to 1.26)	1.75 (1.6 to 1.9)
	Diet high in sugar-sweetened beverages	4.43 (2.02-7.88)	96.29 (44.62-161.94)	96.29 (44.62-161.94)	223.57 (102.19-368.46)	1.8 (1.36 to 2.23)	2.75 (2.44 to 3.07)
	Diet low in fiber	1.01 (0.55-1.55)	23.54 (12.79-35.55)	23.54 (12.79-35.55)	32.19 (16.98-50.40)	0.42 (0.17 to 0.67)	1.04 (0.85 to 1.23)
	Diet low in fruits	2.83 (0.44-5.36)	73.21 (11.28-136.54)	73.21 (11.28-136.54)	69.19 (10.47-131.94)	-0.67 (-0.85 to -0.49)	-0.16 (-0.28 to -0.04)
	Diet low in vegetables	1.21 (-0.41-2.78)	32.38 (-11.77-73.25)	32.38 (-11.77-73.25)	26.64 (-9.68-61.10)	-1.29 (-1.38 to -1.19)	-0.64 (-0.75 to -0.52)
	Diet low in whole grains	33.71 (9.99-54.50)	777.06 (224.69-1268.96)	777.06 (224.69-1268.96)	1180.75 (337.21-1982.83)	0.56 (0.25 to 0.86)	1.36 (1.24 to 1.48)
	Dietary risks	47.16 (12.93-75.91)	1095.53 (294.39-1785.02)	1095.53 (294.39-1785.02)	1749.53 (486.00-2897.88)	0.73 (0.44 to 1.03)	1.53 (1.41 to 1.65)
Oceania	Diet high in processed meat	16.16 (3.57-29.47)	322.54 (70.15-585.91)	19.38 (4.14-36.26)	426.26 (92.49-792.19)	0.58 (0.52 to 0.65)	0.89 (0.83 to 0.96)
	Diet high in red meat	28.39 (-3.99-72.31)	568.05 (-82.34-1428.38)	28.65 (-3.74-71.34)	626.41 (-83.58-1578.45)	0.03 (-0.07 to 0.13)	0.32 (0.24 to 0.4)
	Diet high in sugar-sweetened beverages	9.32 (4.14-16.92)	184.14 (81.18-336.90)	13.25 (5.60-24.74)	288.53 (123.50-534.00)	1.15 (0.98 to 1.32)	1.47 (1.33 to 1.61)
	Diet low in fiber	2.96 (1.48-4.81)	52.96 (27.25-84.20)	2.06 (0.89-3.73)	38.66 (16.94-69.31)	-1.2 (-1.36 to -1.03)	-1.02 (-1.21 to -0.83)
	Diet low in fruits	47.88 (7.41-90.35)	923.95 (142.77-1727.74)	47.29 (6.60-88.43)	992.60 (140.82-1861.74)	-0.07 (-0.12 to -0.02)	0.21 (0.17 to 0.25)
	Diet low in vegetables	23.55 (-8.34-53.57)	426.98 (-153.58-963.62)	16.41 (-5.98-37.21)	322.93 (-118.59-723.35)	-1.18 (-1.34 to -1.03)	-0.92 (-1.03 to -0.8)

	Diet low in whole grains	80.90 (22.45-140.69)	1586.95 (433.47-2732.52)	88.35 (22.60-151.33)	1916.97 (505.36-3269.17)	0.3 (0.23 to 0.36)	0.61 (0.54 to 0.68)
	Dietary risks	182.32 (28.93-321.69)	3550.14 (563.34-6221.14)	190.87 (31.65-333.11)	4094.34 (714.06-7063.38)	0.15 (0.11 to 0.2)	0.46 (0.43 to 0.5)
South Asia	Diet high in processed meat	7.37 (1.72-13.24)	148.57 (33.76-266.40)	10.10 (2.29-17.94)	217.82 (49.92-381.70)	1.13 (0.95 to 1.3)	1.26 (1.03 to 1.5)
	Diet high in red meat	0.93 (-0.12-2.35)	19.94 (-2.59-49.53)	1.37 (-0.18-3.37)	31.11 (-4.30-77.17)	1.29 (0.88 to 1.7)	1.5 (1.38 to 1.61)
	Diet high in sugar-sweetened beverages	1.61 (0.81-2.54)	36.80 (18.38-57.88)	4.51 (2.19-7.04)	104.19 (50.28-161.68)	3.56 (3.18 to 3.93)	3.4 (3 to 3.81)
	Diet low in fiber	3.85 (2.14-5.59)	75.99 (42.61-110.42)	4.03 (2.16-6.17)	81.21 (43.14-124.47)	0.17 (-0.4 to 0.76)	0.23 (0.1 to 0.35)
	Diet low in fruits	19.42 (3.07-35.11)	415.29 (64.01-753.96)	27.10 (3.99-47.53)	592.36 (85.88-1039.41)	1.14 (0.55 to 1.74)	1.17 (0.77 to 1.57)
	Diet low in vegetables	7.11 (-2.49-15.60)	131.84 (-47.99-288.99)	4.19 (-1.46-9.65)	73.98 (-26.76-173.39)	-1.57 (-2.1 to -1.05)	-1.81 (-2.13 to -1.49)
	Diet low in whole grains	12.81 (3.81-21.61)	270.97 (79.39-461.52)	15.35 (4.16-25.73)	341.53 (92.70-569.91)	0.59 (0.21 to 0.98)	0.76 (0.62 to 0.89)
	Dietary risks	43.91 (8.01-74.91)	915.24 (172.27-1578.15)	56.95 (11.90-95.10)	1238.52 (269.66-2060.49)	0.89 (0.34 to 1.45)	1.04 (0.9 to 1.18)
Southeast Asia	Diet high in processed meat	3.35 (0.77-5.97)	80.93 (18.05-143.07)	5.49 (1.19-9.58)	154.02 (33.52-273.33)	1.65 (1.53 to 1.77)	2.09 (1.99 to 2.19)
	Diet high in red meat	2.37 (-0.31-5.86)	55.37 (-7.77-133.16)	5.83 (-0.81-14.11)	156.56 (-22.89-379.15)	2.96 (2.79 to 3.12)	3.41 (3.31 to 3.52)
	Diet high in sugar-sweetened beverages	1.58 (0.76-2.64)	38.39 (18.59-63.63)	4.91 (2.29-8.04)	139.49 (65.01-225.27)	3.74 (3.48 to 3.99)	4.29 (4.07 to 4.51)
	Diet low in fiber	7.10 (4.03-10.17)	160.21 (90.75-229.99)	6.23 (3.48-9.15)	160.09 (86.76-234.71)	-0.43 (-0.48 to -0.37)	-0.02 (-0.07 to 0.03)
	Diet low in fruits	14.09 (2.22-25.21)	323.23 (50.01-574.47)	10.29 (1.57-18.76)	267.28 (40.47-484.69)	-1.01 (-1.08 to -0.93)	-0.62 (-0.71 to -0.54)
	Diet low in vegetables	6.70 (-2.53-15.40)	151.60 (-57.82-345.85)	1.39 (-0.46-3.41)	35.64 (-12.32-87.58)	-4.99 (-5.18 to -4.81)	-4.62 (-4.85 to -4.39)
	Diet low in whole grains	4.15 (1.15-7.26)	97.47 (26.73-168.33)	4.30 (1.15-7.43)	118.45 (31.79-207.56)	0.1 (-0.03 to 0.23)	0.63 (0.54 to 0.73)
	Dietary risks	30.22 (3.34-54.75)	700.07 (78.87-1261.72)	30.51 (4.88-54.36)	824.97 (145.11-1491.71)	0.04 (-0.11 to 0.19)	0.52 (0.44 to 0.59)
Southern Latin America	Diet high in processed meat	22.07 (5.10-38.61)	513.82 (118.31-899.60)	19.32 (4.68-32.25)	682.36 (157.70-1195.34)	-0.41 (-0.88 to 0.06)	0.93 (0.61 to 1.24)
	Diet high in red meat	19.26 (-3.36-41.71)	437.57 (-82.19-961.24)	13.94 (-2.34-30.19)	472.72 (-90.45-1068.19)	-0.99 (-1.48 to -0.5)	0.28 (-0.03 to 0.58)

	Diet high in sugar-sweetened beverages	12.55 (5.51-21.22)	287.89 (129.06-482.46)	12.05 (5.82-18.88)	415.79 (192.47-679.27)	-0.17 (-0.52 to 0.19)	1.23 (0.97 to 1.48)
	Diet low in fiber	2.98 (1.64-4.36)	64.39 (35.76-94.37)	1.81 (0.95-2.76)	58.07 (30.02-92.16)	-1.6 (-2.29 to -0.9)	-0.34 (-0.75 to 0.06)
	Diet low in fruits	4.41 (0.67-8.41)	98.47 (14.58-187.40)	1.53 (0.23-3.28)	50.03 (7.16-107.89)	-3.49 (-4.28 to -2.7)	-2.24 (-2.64 to -1.83)
	Diet low in vegetables	0.51 (-0.16-1.22)	10.52 (-3.51-25.17)	0.22 (-0.07-0.50)	6.90 (-2.35-16.48)	-2.78 (-3.23 to -2.32)	-1.35 (-1.67 to -1.03)
	Diet low in whole grains	32.10 (9.25-52.11)	720.47 (208.97-1186.94)	16.22 (4.46-26.57)	522.29 (140.81-913.25)	-2.16 (-2.81 to -1.51)	-1.03 (-1.46 to -0.59)
	Dietary risks	73.10 (16.02-113.59)	1658.32 (388.33-2609.88)	50.99 (12.20-78.30)	1727.80 (422.61-2806.85)	-1.12 (-1.59 to -0.65)	0.15 (-0.13 to 0.44)
Southern Sub-Saharan Africa	Diet high in processed meat	14.31 (3.42-26.00)	298.77 (71.21-534.09)	32.71 (7.87-60.08)	710.21 (170.12-1298.81)	2.72 (2.29 to 3.15)	2.86 (2.52 to 3.21)
	Diet high in red meat	14.55 (-2.09-34.17)	304.63 (-44.72-710.40)	31.41 (-4.21-76.41)	675.37 (-94.64-1635.04)	2.54 (2.08 to 2.99)	2.61 (2.23 to 3)
	Diet high in sugar-sweetened beverages	8.75 (3.52-16.70)	178.45 (72.95-335.61)	23.25 (9.61-43.26)	490.88 (206.07-879.76)	3.22 (2.72 to 3.73)	3.35 (2.96 to 3.74)
	Diet low in fiber	2.00 (1.03-3.22)	38.78 (20.21-61.24)	3.97 (1.79-6.87)	79.54 (36.49-135.38)	2.27 (1.91 to 2.63)	2.35 (2.04 to 2.65)
	Diet low in fruits	28.22 (4.49-49.50)	573.42 (89.76-1010.95)	51.78 (7.98-92.26)	1070.81 (163.40-1938.79)	2 (1.54 to 2.46)	2.05 (1.66 to 2.44)
	Diet low in vegetables	11.43 (-4.13-25.18)	231.29 (-84.27-507.96)	13.40 (-4.93-31.82)	281.84 (-104.91-645.41)	0.6 (0.25 to 0.95)	0.66 (0.47 to 0.85)
	Diet low in whole grains	6.11 (1.63-11.17)	123.63 (33.23-220.78)	10.49 (2.79-20.41)	217.09 (57.64-411.70)	1.76 (1.37 to 2.15)	1.83 (1.5 to 2.17)
	Dietary risks	74.54 (9.19-130.28)	1527.61 (186.56-2666.86)	145.41 (22.59-251.05)	3066.48 (468.30-5318.48)	2.2 (1.76 to 2.64)	2.28 (1.92 to 2.64)
Tropical Latin America	Diet high in processed meat	10.90 (2.41-20.14)	260.72 (56.27-481.41)	13.99 (3.23-25.77)	395.84 (91.79-730.20)	0.85 (0.5 to 1.2)	1.37 (1.23 to 1.51)
	Diet high in red meat	15.98 (-2.17-36.73)	377.34 (-52.87-855.84)	21.46 (-3.33-47.59)	587.44 (-99.12-1312.22)	1.02 (0.52 to 1.52)	1.47 (1.29 to 1.66)
	Diet high in sugar-sweetened beverages	7.35 (2.93-14.27)	175.99 (71.76-329.59)	10.49 (4.48-18.61)	285.53 (124.81-495.43)	1.15 (0.72 to 1.59)	1.63 (1.43 to 1.84)
	Diet low in fiber	3.74 (2.02-5.57)	82.64 (45.08-121.34)	2.31 (1.13-3.83)	58.27 (28.79-95.38)	-1.44 (-2.13 to -0.74)	-1.05 (-1.41 to -0.68)
	Diet low in fruits	5.51 (0.87-11.15)	123.01 (19.66-244.13)	2.58 (0.34-6.40)	60.53 (7.90-149.79)	-2.37 (-2.59 to -2.14)	-2.25 (-2.42 to -2.08)
	Diet low in vegetables	5.02 (-1.58-12.61)	105.22 (-35.07-255.93)	1.04 (-0.32-3.18)	24.06 (-7.37-70.62)	-4.95 (-5.22 to -4.68)	-4.65 (-4.86 to -4.45)

	Diet low in whole grains	12.59 (3.46-22.07)	290.82 (80.72-504.90)	7.92 (2.12-15.13)	204.27 (55.55-385.51)	-1.46 (-2.03 to -0.89)	-1.09 (-1.44 to -0.75)
	Dietary risks	51.59 (6.95-91.56)	1198.54 (163.63-2130.20)	51.41 (6.79-90.09)	1388.95 (189.88-2467.81)	0.01 (-0.51 to 0.53)	0.5 (0.28 to 0.72)
Western Europe	Diet high in processed meat	25.14 (6.00-40.80)	530.73 (126.80-877.35)	14.67 (3.48-23.94)	495.39 (114.66-851.35)	-1.7 (-1.85 to -1.54)	-0.22 (-0.34 to -0.09)
	Diet high in red meat	11.86 (-1.72-26.16)	256.85 (-39.80-571.72)	6.74 (-0.99-14.98)	229.92 (-39.47-531.65)	-1.79 (-1.94 to -1.65)	-0.36 (-0.48 to -0.24)
	Diet high in sugar-sweetened beverages	6.28 (3.04-9.86)	133.12 (65.37-207.70)	4.41 (2.15-6.80)	156.33 (75.24-250.08)	-1.12 (-1.3 to -0.93)	0.52 (0.44 to 0.6)
	Diet low in fiber	1.57 (0.87-2.30)	31.07 (17.17-45.40)	0.87 (0.46-1.29)	27.15 (14.19-42.11)	-1.9 (-2.21 to -1.58)	-0.44 (-0.6 to -0.26)
	Diet low in fruits	2.79 (0.46-5.08)	55.87 (8.92-103.58)	1.43 (0.22-2.76)	47.38 (7.55-91.93)	-2.12 (-2.4 to -1.84)	-0.54 (-0.7 to -0.38)
	Diet low in vegetables	0.19 (-0.06-0.45)	3.80 (-1.26-8.86)	0.11 (-0.04-0.26)	3.53 (-1.15-8.49)	-1.8 (-2.04 to -1.55)	-0.24 (-0.41 to -0.06)
	Diet low in whole grains	13.83 (3.99-22.54)	296.37 (85.83-494.53)	8.94 (2.55-14.69)	284.92 (80.69-486.21)	-1.41 (-1.54 to -1.27)	-0.16 (-0.32 to 0)
	Dietary risks	49.79 (10.65-77.85)	1054.80 (236.21-1675.15)	29.81 (6.70-46.48)	996.42 (240.89-1635.22)	-1.61 (-1.75 to -1.46)	-0.19 (-0.32 to -0.07)
Western Sub-Saharan Africa	Diet high in processed meat	23.06 (5.68-40.68)	479.06 (115.51-847.37)	34.02 (8.53-58.67)	766.15 (185.21-1334.85)	1.26 (1.21 to 1.32)	1.52 (1.47 to 1.58)
	Diet high in red meat	2.98 (-0.41-7.22)	63.85 (-9.17-152.25)	4.91 (-0.66-11.97)	114.42 (-16.09-278.07)	1.63 (1.58 to 1.68)	1.91 (1.85 to 1.97)
	Diet high in sugar-sweetened beverages	0.97 (0.43-1.80)	19.65 (8.84-36.37)	3.22 (1.40-5.87)	70.88 (30.71-127.93)	3.97 (3.75 to 4.18)	4.26 (4.15 to 4.38)
	Diet low in fiber	1.71 (0.92-2.63)	32.84 (17.87-49.35)	0.94 (0.46-1.55)	18.72 (9.29-30.50)	-1.9 (-2.05 to -1.76)	-1.78 (-1.93 to -1.64)
	Diet low in fruits	14.10 (2.31-25.51)	281.08 (44.59-502.28)	16.23 (2.54-29.14)	353.67 (54.34-638.17)	0.46 (0.38 to 0.54)	0.75 (0.69 to 0.8)
	Diet low in vegetables	6.21 (-2.43-14.00)	120.24 (-46.56-263.08)	5.36 (-1.98-12.27)	120.74 (-46.51-277.35)	-0.47 (-0.56 to -0.38)	0.02 (-0.09 to 0.12)
	Diet low in whole grains	20.33 (5.88-34.16)	409.17 (118.73-686.42)	20.08 (5.66-34.05)	429.77 (121.23-738.42)	-0.03 (-0.11 to 0.05)	0.16 (0.07 to 0.24)
	Dietary risks	60.30 (13.00-100.77)	1221.73 (265.37-2022.71)	75.13 (16.58-123.65)	1658.66 (360.45-2774.99)	0.72 (0.66 to 0.78)	0.99 (0.95 to 1.04)

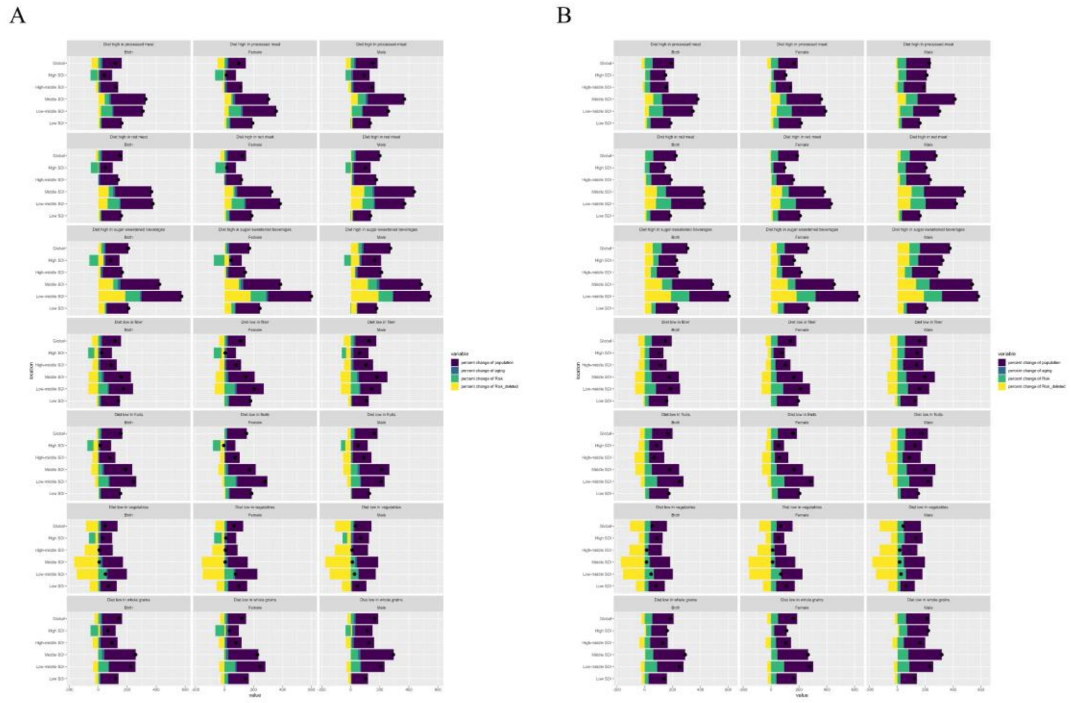

Supplement 3. Decomposition of changes in the burden of T2DM attributable to dietary risk factors among elderly adults across SDI levels, 1990–2021

A. Mortality: The percent changes in T2DM-related deaths attributable to dietary risk factors are decomposed into four components: population growth (purple), population aging (green), changes in dietary risk exposure (yellow), and other residual factors, including risk-deleted mortality (blue). Results are stratified by global, high SDI, high-middle SDI, middle SDI, low-middle SDI, and low SDI regions.

B. DALYs: The percent changes in T2DM-related DALYs attributable to dietary risk factors are similarly decomposed into the same four components and stratified across SDI regions.

Legend:

- The chart highlights the differential contributions of demographic changes and dietary risk exposures to the T2DM burden across varying socio-demographic contexts.
- Population growth and aging emerge as dominant drivers in low and low-middle SDI regions, while dietary risk exposure plays a more significant role in middle and high SDI regions. The results emphasize the interplay of demographic and behavioral factors in shaping the burden of T2DM.
